# HPV 5 and 8 E6 Abrogate ATR Activity Resulting in Increased Persistence of UVB Induced DNA Damage

**DOI:** 10.1371/journal.ppat.1002807

**Published:** 2012-07-12

**Authors:** Nicholas A. Wallace, Kristin Robinson, Heather L. Howie, Denise A. Galloway

**Affiliations:** Division of Human Biology, Fred Hutchinson Cancer Research Center, Seattle, Washington, United States of America; University of Virginia, United States of America

## Abstract

The role of the E6 oncoprotein from high-risk members of the α human papillomavirus genus in anogenital cancer has been well established. However, far less is known about the E6 protein from the β human papillomavirus genus (β-HPVs). Some β-HPVs potentially play a role in non-melanoma skin cancer development, although they are not required for tumor maintenance. Instead, they may act as a co-factor that enhances the carcinogenic potential of UV damage. Indeed, the E6 protein from certain β-HPVs (HPV 5 and 8) promotes the degradation of p300, a histone acetyl transferase involved in UV damage repair. Here, we show that the expression of HPV 5 and 8 E6 increases thymine dimer persistence as well as the likelihood of a UVB induced double strand break (DSB). Importantly, we provide a mechanism for the increased DNA damage by showing that both extended thymine dimer persistence as well as elevated DSB levels are dependent on the ability of HPV 8 E6 to promote p300 degradation. We further demonstrate that HPV 5 and 8 E6 expression reduces the mRNA and protein levels of ATR, a PI3 kinase family member that plays a key role in UV damage signaling, but that these levels remain unperturbed in cells expressing a mutated HPV 8 E6 incapable of promoting p300 degradation. We confirm that the degradation of p300 leads to a reduction in ATR protein levels, by showing that ATR levels rebound when a p300 mutant resistant to HPV 8 mediated degradation and HPV 8 E6 are co-transfected. Conversely, we show that ATR protein levels are reduced when p300 is targeted for degradation by siRNA. Moreover, we show the reduced ATR levels in HPV 5 and 8 E6 expressing cells results in delayed ATR activation and an attenuated ability of cells to phosphorylate, and as a result accumulate, p53 in response to UVB exposure, leading to significantly reduced cell cycle arrest. In conclusion, these data demonstrate that β-HPV E6 expression can enhance the carcinogenic potential of UVB exposure by promoting p300 degradation, resulting in a reduction in ATR levels, which leads to increased thymine dimer persistence and increased UVB induced DSBs.

## Author Summary

Human papillomaviruses are a large family of viruses that can cause ailments ranging from benign warts to anogenital cancer. Recently, interest has increased for a subgroup of these viruses, the β-HPVs, because of their potential involvement in squamous cell skin cancer. In this work, we show that the E6 protein from two of these viruses (HPV 5 and 8) is able to increase the damage that UV exposure causes to the host cell's DNA. The E6 protein from these viruses promotes the degradation of p300, a cellular protein involved in DNA damage repair. This in turn reduces amounts of another cellular protein, ATR, which facilitates the process of signaling the cell to repair its damaged DNA. The decrease in ATR levels delays the cell's recognition of the damaged DNA, allowing thymine dimers to remain unrepaired longer and more often leading to a double strand break in the DNA. Together, our data show that the β HPVs can cause UV exposures to be more deleterious to host cell DNA, potentially increasing the likelihood that these cells become cancerous.

## Introduction

Human papillomaviruses (HPV) are a large family of double stranded DNA viruses that infect the cutaneous and mucosal epidermis of humans. This family of viruses is divided, based on DNA sequence homology, into 5 genera [1]. Of these genera, alpha human papillomaviruses (α-HPV) are the most commonly studied due to the association of some α-HPVs with anogenital cancers [2], [3]. Members of the α-HPV genus include both low risk (HPV types 6 and 11) and high risk (HPV types 16 and 18) types, designated to denote their likelihood of inducing a carcinoma. While low risk α-HPV (LR α-HPV) infections are most often associated with more benign conditions such as genital warts, high risk α-HPV (HR α-HPV) infections have been established as the causative agent of nearly all cervical and a subset of head and neck cancers [2]–[4]. Recently, members of the β human papillomavirus (β-HPV) genus (particularly HPV types 5 and 8) have gained increasing interest due to a potential association with non-melanoma skin cancer (NMSC) [5]–[9]. Since the β-HPV viruses do not seem to be necessary for tumor maintenance, they may act as co-factors to increase the mutagenic potential of UV exposure [8], [10].

Most HPV genera express 8 genes, which are categorized as either early (E) or late (L), based on when they are expressed during the viral life cycle. Two early HPV gene products, HPV E6 and HPV E7, are particularly well characterized and considered the primary oncogenes in HR α-HPVs [11], [12]. Some functions of the E6 protein are conserved between HR and LR α-HPVs, such as the ability to bind E6AP and degrade the pro-apoptotic Bak protein [13]–[18]. However, the ability to activate telomerase, degrade p53, and associate with multiple PDZ domain containing proteins functionally differentiates these two classes of E6 proteins [19]–[28]. Not surprisingly, the LR and HR α-HPV E6 proteins have sequence heterogeneity associated with these functional differences such as the presence of a PDZ binding domain in HR α-HPV E6 that is absent in LR α-HPV E6 proteins [29].

Unlike the α-HPV E6 proteins, considerably less is known about β-HPV E6 proteins. Similar to α-HPVs, β-HPV E6 proteins are capable of binding to and degrading Bak [30], [31]. Additionally, like HR α-HPV E6 proteins, β-HPV E6 proteins can bind to the cellular histone acetyl transferase (HAT), p300 [32]–[35]. Some β-HPV E6 proteins bind p300 more strongly, blocking phosphorylation by AKT, resulting in destabilization of p300 [36]. Finally, like the LR α-HPV E6s, β-HPV E6 proteins lack a PDZ binding domain and as a result are unable to target PDZ domain proteins for degradation.

Perhaps the biggest difference between HR α-HPV E6 and β-HPV E6 proteins is the ability of HR α-HPV E6 protein to degrade p53, a function lacking in β-HPV E6 proteins. Instead, the β-HPV 38 E6 can instead attenuate p53 signaling by inducing the expression of deltaNp73 [37], a dominant negative inhibitor of p53. Additionally HPV 5 and 8 E6 promote the degradation of p300 potentially abrogating its ability to acetylate p53 in response to UV damage [38]. Furthermore, β-HPV E6 expression has been shown to result in persistence of thymine dimers following UVB exposure [39].

Typically in response to UVB exposure a PI3 kinase family member, ATR, phosphorylates p53, blocking MDM2 mediated proteosomal degradation and allowing p53 to accumulate and activate genes involved in DNA damage repair as well as cell cycle arrest [40]–[46]. p300 associates with ATR and its acetyl transferase activity is required for ATR to phosphorylate one of its downstream kinase targets, CHK1, providing evidence for a functional relationship between p300 and ATR [47]. In this work, we report that cells expressing HPV 5 and 8 E6 proteins that are capable of promoting p300 degradation have reduced ATR levels and activation, while ATR levels are unperturbed in cells expressing HPV Δ8 E6, an E6 mutant that cannot promote p300 degradation. We provide additional support for the connection between ATR and p300 by demonstrating that ATR levels are similar to control levels when cells are co-transfected with HPV 8 E6 and a mutated p300 resistant to HPV 8 E6 promoted degradation. Conversely, ATR levels are reduced in cells transfected with p300 targeting siRNA.

The reduction in ATR expression attenuates ATR activity resulting in decreased phosphorylation of p53. Reduced p53 phosphorylation delays p53 accumulation following UVB exposure. We also show that β-HPV E6 expression leads to reduced G1 arrest following UVB exposure, allowing more cells to enter S-phase with damaged DNA. The collision of a replication fork with a bulky thymine dimer can cause the fork to collapse into a deleterious double stranded break (DSB) in DNA [48]. Importantly, we demonstrate that β-HPV E6 expression increases the likelihood of DSB formation following UVB exposure. In HPV 5 and 8 E6 expressing cells, UVB induced DSBs persist for significantly longer and occur approximately four times more frequently. Finally, the decreased ability of β-HPV E6 expressing cells to repair UVB induced DNA damage results in increased sensitivity to UVB.

## Results

### β HPV E6 expression increases thymine dimer persistence and DSB levels following UVB

**Figure 1:**
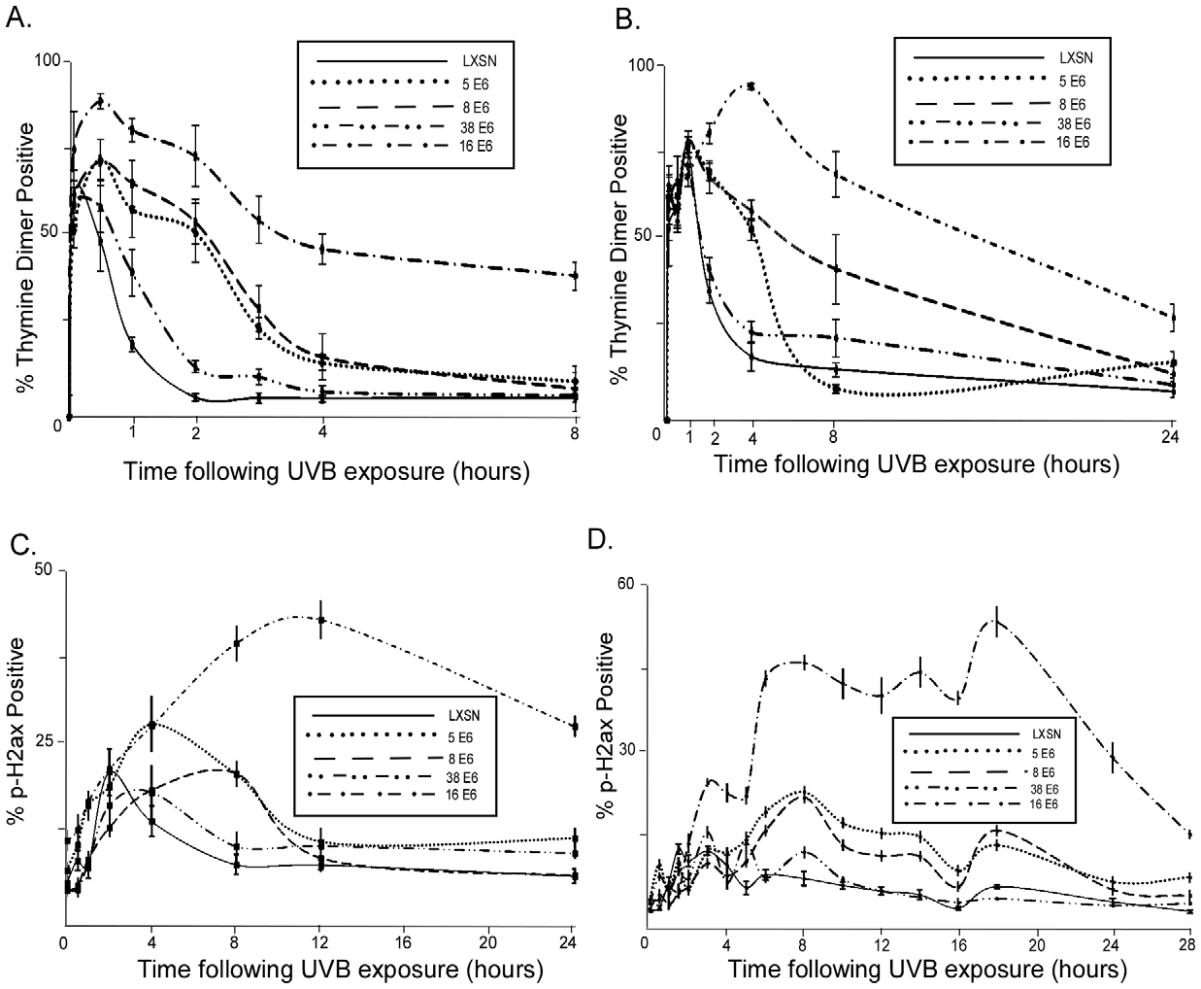
HPV E6 increases thymine dimer persistence and DSB prevalence following UVB exposure. A. HFK cells were exposed to 10 mJ/cm^2^ UVB and immunofluorescence was used to measure the percentage of cells positive for thymine dimers above background. B. HT1080 cells were exposed to 10 mJ/cm^2^ UVB and immunofluorescence was used to measure the percentage of cells positive for thymine dimers above background. C. HFK cells were exposed to 10 mJ/cm^2^ UVB and immunofluorescence was used to measure the percentage of cells positive for phospho H2AX above background. D. HT1080 cells were exposed to 10 mJ/cm^2^ UVB and immunofluorescence was used to measure the percentage of cells positive for phospho H2AX above background. For all portions of this figure, n≥5, error bars represent standard errors of the mean.

UVB induced thymine dimer repair has previously been shown to be significantly delayed in cells expressing HPV 5 E6 and HR α-HPV 18 E6 [39]. To determine if the expression of other HPV E6 proteins could also increase thymine dimer persistence, we first expressed 3 β-HPV E6 (5, 8, and 38 E6) and 1 HR α-HPV E6 (16 E6) in human foreskin keratinocytes (HFK) cells and HT1080 cells. HPV E6 expression was confirmed using semi quantitative RT-PCR (Figure S1). We examined the cells for the presence of thymine dimers by immunofluorescent microscopy and quantitated the number of cells staining positive for thymine dimers above background and were able to confirm that HPV 5 E6 expression resulted in increased persistence of thymine dimers [39] and showed that HPV 8 and 16 E6 expression also resulted in this phenotype (Figure 1A, 1B, and Figure S2). Indeed, although the peak number of cells positive for thymine dimer occurred rapidly after UVB exposure (less than one hour after exposure) and was similar among the cell lines, a significantly higher percentage of HPV 5, 8, and 16 E6 expressing cells maintained elevated thymine dimer levels three hours after UVB exposure. Interestingly, thymine dimer persistence in HPV 38 E6 expressing cells was most similar to vector control cells. Thymine dimers persisted far longer in HPV 16 E6 expressing HFK and HT1080 cells than the other cell lines. Over 40% of 16 E6 expressing HFKs and over 25% of HPV 16 E6 expressing HT1080 cells were positive for thymine dimers after all other cells lines returned to background thymine dimer levels (8 hours for HFKs, and 12 hours for HT1080 cells). Notably, thymine dimers persisted far longer in HT1080 cells than in HFK cells, perhaps due to the transformed nature of HT1080 cells.

To determine whether persistence of thymine dimers in β-HPV E6 expressing cells was due to an increased number of thymine dimers forming in response to the same dose of UVB, we quantified thymine dimer formation directly using antibody specific for the lesion in response to a gradient of UVB exposures ranging from 0 to 25 mJ/cm^2^. Thymine dimer formation varied minimally between HPV E6 expressing cells lines and control cells, (Figure S3) indicating that altered thymine dimer formation did not explain the substantially increased persistence of thymine dimers in HPV 5, 8, and 16 E6 expressing cells. Since nucleotide excision repair (NER) is the DNA damage repair (DDR) pathway responsible for the repair of UVB induced thymine dimers [49], these data suggest that NER is attenuated in HPV 5, 8, and 16 E6 expressing cells.

Persistent thymine dimers can result in the collapse of replication forks into double strand breaks (DSBs) if they remain unrepaired as a cell progresses through S phase [48]. We therefore hypothesized that the increased longevity of thymine dimers in β-HPV E6 expressing cells would result in increased UV induced DSB formation in these cells. To test this hypothesis, we measured levels of phosphorylated histone H2AX, a commonly used marker of DSBs [50], in HPV E6 expressing cells following UV exposure. Importantly, in both HFK and HT1080 cells expressing HPV 5, 8, and 16 E6 significantly more cells showed evidence of a DSB than vector control cells (Figure 1C, 1D and Figure S4). Also, this increased damage persisted for significantly longer. Vector control cells peaked with approximately 15% of the cells positive for phosphorylated H2AX 1.5 hours after UVB exposure and these levels returned to background by 16 hours. Despite having similar baseline levels of H2AX phosphorylation (data not shown), HPV 5 and 8 E6 expressing cells remained at peak levels, approximately 20% of cells staining positive for phosphorylated H2AX, 8 hours after UVB exposure. HPV 5 and 8 E6 expressing cells maintained elevate levels for over 24 hours following UVB exposure. A greatly increased number of HPV 16 E6 cells had phosphorylated H2AX 12 hours after UVB exposure, with approximately 40% of the cells positive. The levels of phosphorylated H2AX also persist longer in HPV 16 E6 expressing cells. 24 hours after UVB exposure, HPV 16 E6 expressing cells had a high percentage of cells staining positive for phosphorylated H2AX than control cells experience at their peak levels. Similar to what we observed when measuring thymine dimers following UV exposure, HPV 38 E6 expressing HT1080 cells had more cells with DSBs than vector control cells at 3 hours in HT1080 cells and at 4 hours in HFK cells after UVB exposure, but they were more similar to vector control cells than HPV 5, 8, or 16 E6 expressing cells (Figure 1C, D and Figure S4).

**Figure 2:**
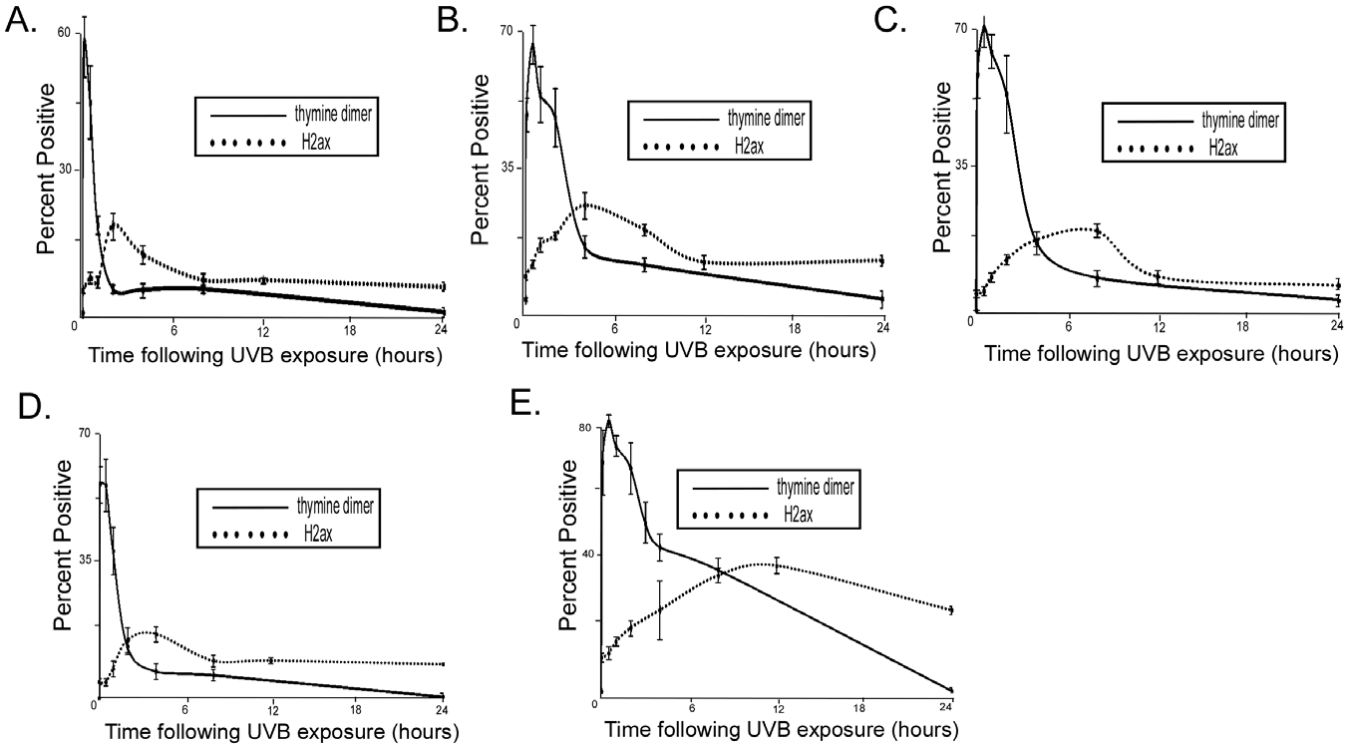
Temporal relationship between thymine dimer and phospho H2AX detection. HFK cells were exposed to 10 mJ/cm^2^ UVB and immunofluorescence was used to measure the percentage of cells positive for thymine dimers and phospho H2AX above background. Graph shows percentage of HFK cells staining positive for either thymine dimers (solid line) or phospho H2AX (dotted line) after indicated period of time following exposure to 10 mJ/cm^2^ UVB. A. LXSN HFK cells B. 5 E6 HFK cells C. HPV 8 E6 HFK cells D. HPV 38 E6 HFK cells E. HPV 16 E6 HFK cells. n≥5. Error bars represent standard errors of the mean.

To determine if UVB induced phosphorylation of H2AX was consistent with a model where DSBs occur following UVB exposure as a result of replication fork collapse following collision with an unrepaired thymine dimer, we compared the temporal relationship between thymine dimer formation and phospho H2AX staining. For each cell line we tested, the peak number of cells staining positive for thymine dimers occurred either before or at the same time as the peak number of cells positive for phospho-H2AX (Figure 2). These data suggest that while thymine dimers can occur either before or at the same time as UVB induced DSBs, UVB induced double strand breaks do not occur before the formation of thymine dimers.

### β HPV E6 mediated increased thymine dimer persistence and elevated DSB levels in cells following UVB exposure is dependent on p300 degradation

**Figure 3:**
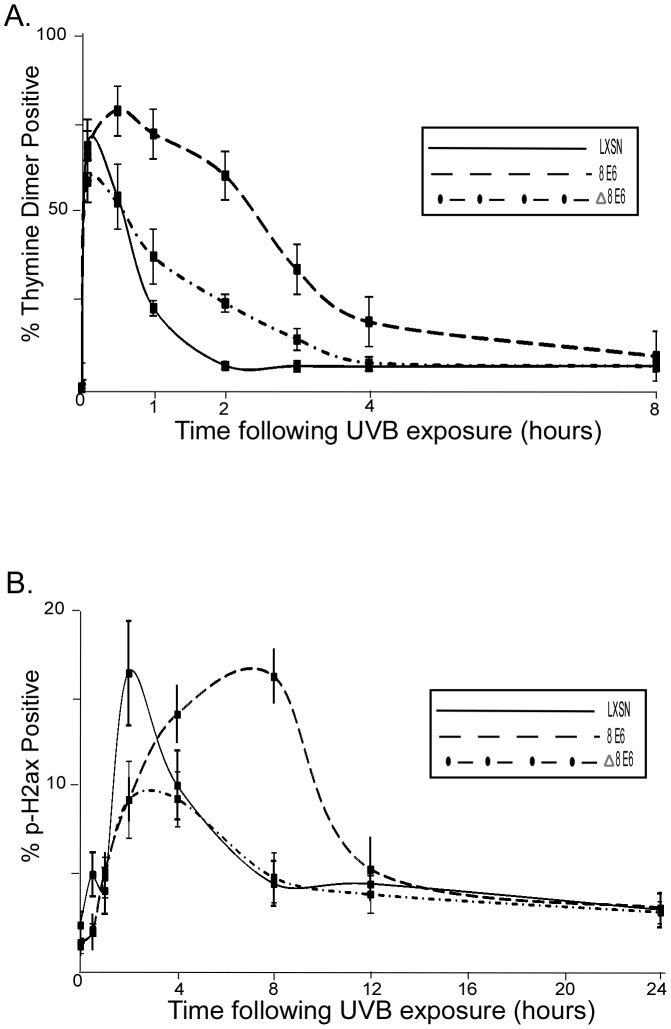
Increased persistence of UVB induced DNA damage in HPV 8 E6 expressing cells is dependent on p300 degradation. A. HFK cells were exposed to 10 mJ/cm^2^ UVB and immunofluorescence was used to measure the percentage of cells positive for thymine dimers above background. B. HFK cells were exposed to 10 mJ/cm^2^ UVB and immunofluorescence was used to measure the percentage of cells positive for phospho H2AX above background. For all portions of this figure, n≥5, error bars represent standard errors of the mean, and Δ8 E6 denotes cells expressing a mutant form of 8 E6 incapable of promoting p300 degradation.

HPV 5 and 8 E6 promote the degradation of p300 [36], a key HAT involved in UVB damage response [51]–[53]. Indeed, p300 levels were reduced in HPV 5 and 8 E6 expressing HFK cells (data not shown) that had elevated thymine dimer persistence and phospho-H2AX levels following UVB exposure. To determine if the increase in thymine dimer persistence and phospho H2AX levels in these cell lines was related to their ability to degrade p300, we measured thymine dimer persistence and phospho H2AX levels in HFK cells expressing a mutant of HPV 8 E6 (HPV Δ8 E6) that is incapable of degrading p300 but maintains other functions such as its ability to activate the HPV 8 promoter [32]. Thymine dimer removal in HPV Δ8 E6 expressing cells was not significantly different from vector control HFK cells (Figure 3A). Phospho H2AX levels in Δ8 E6 HFK cells were also similar to the levels seen in cells with normal p300 levels (Figure 3B).

### β HPV E6 expression increases sensitivity to crosslinking agents

**Figure 4:**
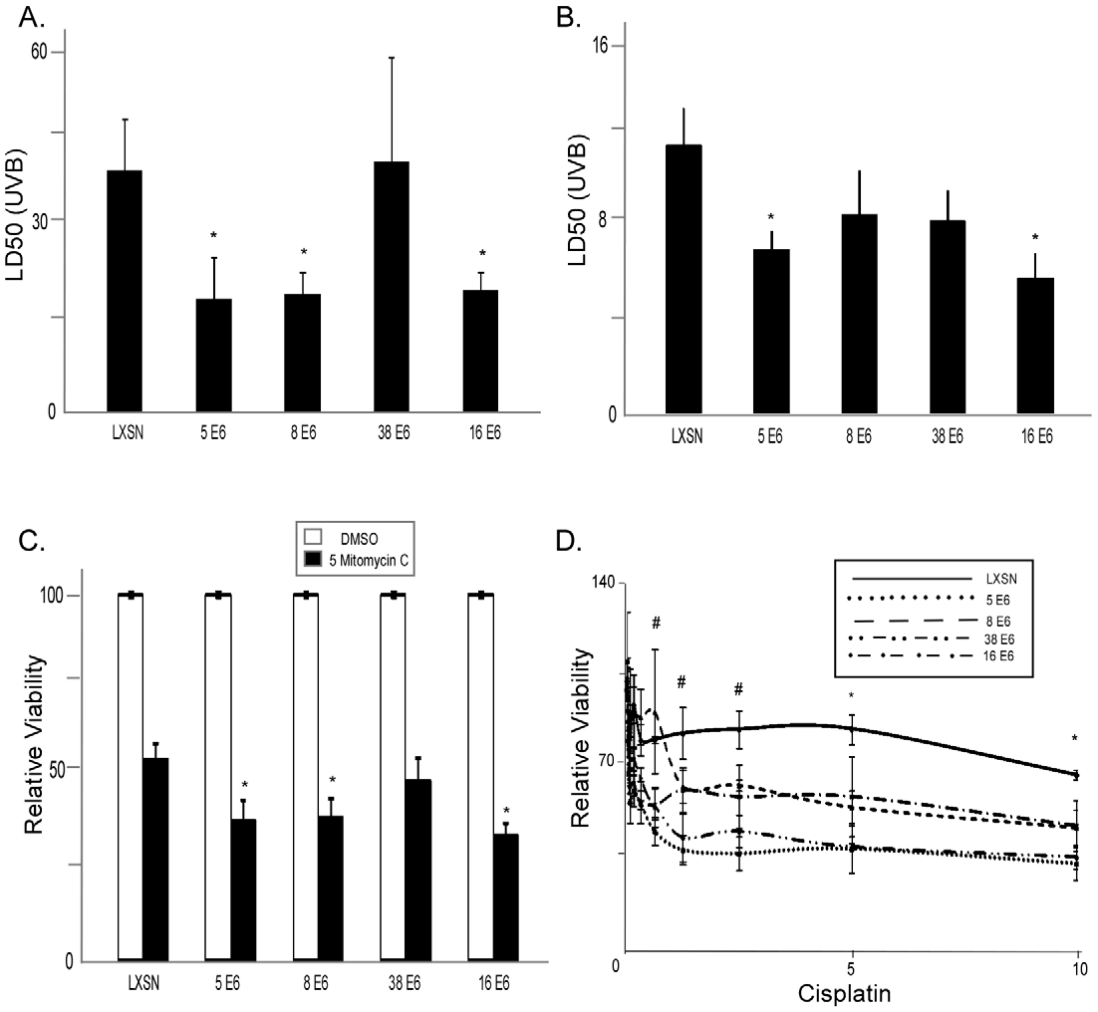
HPV E6 expression increases sensitivity to crosslinking agents. A. Median Lethal dose of UVB for HPV E6 expressing primary human foreskin keratinocytes (HFKs) was measured following exposure to a range of UVB doses (0–20 mJ/cm^2^). n≥5. Error bars represent standard errors of the mean. B. Median Lethal dose of UVB for HT1080 cells expressing the same HPV E6s was measured following a range of UVB doses (0–10 mJ/cm^2^). n≥5. Error bars represent standard errors of the mean. C. Relative viability of HPV E6 expressing HT1080 cells was measured after exposure to mitomycin C (µM). n≥5. Error bars represent standard errors of the mean. D. Relative viability was measured in HPV E6 expressing HT1080 cells following exposure to a range of cisplatin doses (µM). n≥5. Error bars represent standard errors of the mean. Linear regression lines were calculated using GraphPad Prism software. # denotes a statistical significant difference from LXSN for HPV 5, 38, and 16 E6 expressing cells (p≤0.05). * denotes a statistical significant difference from LXSN for all HPV E6 expressing cells (p≤0.05).

We have shown that NER is disrupted in HPV 5, 8, and 16 E6 expressing cells (Figure 1A, B). Since NER is responsible for the repair of UVB induced DNA lesions, we hypothesized that UVB exposure would be more deleterious in cells expressing these HPV E6s. In these cells, we determined cellular viability by measuring the number of cells 3 days following exposure to a gradient of UVB doses. To compare the sensitivity to UVB exposure among the cell lines, we used linear regression analysis to determine the median lethal dose (LD50) of UVB exposure (Figure S5). The LD50 of UVB exposure for HPV 5, 8, and 16 E6 expressing HFK cells [19.0+/−7.1 mJ/cm^2^, 19.9+/−3.8 mJ/cm^2^, and 20.6+/−3.1 mJ/cm^2^), respectively] were lower than vector control HFK cells [40.7+/−8.8 mJ/cm^2^)] (Figure 4A, Figure S5). The increased sensitivity was not limited to HFKs as similar results were observed in HT1080 cells expressing HPV 5 and 16 E6 (Figure 4B). Interestingly, perhaps due to the transformed nature of the cells, HT1080 cells not only repair UVB damage more slowly, but also are more sensitive to UVB exposure than HFK cells.

NER is responsible not only for repair of UVB induced lesions, but also the repair of other bulky lesions like the ones induced by chemotherapeutic crosslinking drugs, such as cisplatin and mitomycin C [54]. We hypothesized that if E6 expression leads to the disruption of NER then there will be a general increased sensitivity to DNA crosslinking agents. To test this hypothesis, we measured the viability of HT1080 cells expressing HPV 5, 8, 38, or 16 E6 in the presence of cisplatin and mitomycin C. Consistent with our hypothesis, HPV 5, 8, and 16 E6 expressing cells were also significantly more sensitive to both cisplatin and mitomycin C than vector control cells (Figure 4 C and D). HPV 38 E6 expressing HT1080 cells were more significantly more sensitive to cisplatin, but the increased sensitivity did not reach statistical significance for mitomycin C exposure (Figure 4C and D).

### β HPV E6 expression attenuates ATR expression by promoting p300 degradation

**Figure 5:**
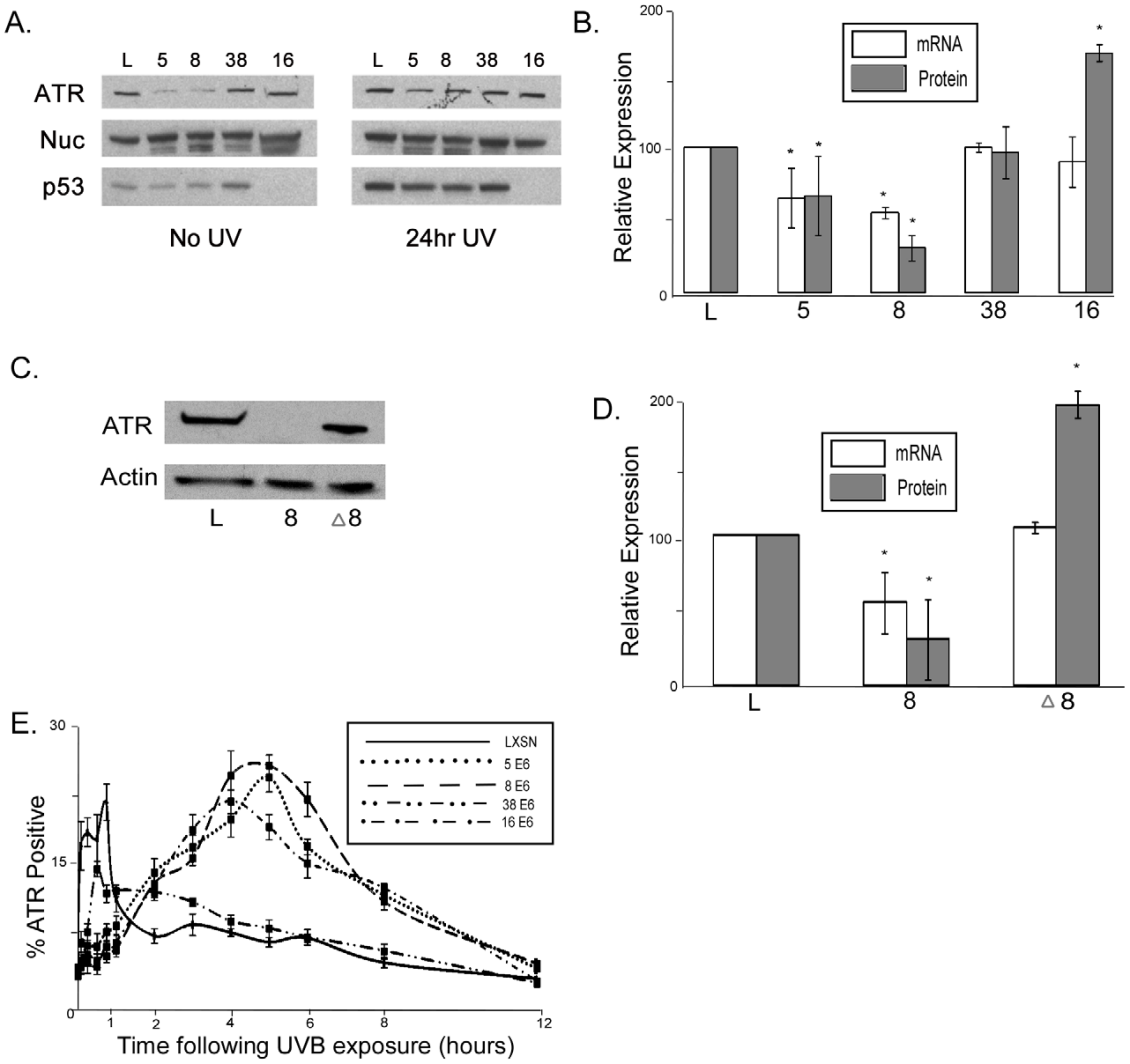
HPV E6 expression decreases ATR expression and activity. A. Representative immunoblot showing ATR protein levels in HFK cells either untreated or exposed to 10 mJ/cm^2^ UVB. p53 is shown as a control for HPV 16 E6 expression, which results in p53 degradation. Nuc denotes Nucleolin. B. Densitometry of immunoblots (n = 3) and measurements by quantitative RT-PCR of ATR mRNA levels (n = 3) in HPV E6 expressing HFK cells. Levels were normalized to GAPdh (mRNA) and Nucleolin (protein) for each experiment. Error bars represent standard errors of the mean. * denotes a statistically significant difference from LXSN cells. C. Representative immunoblot showing ATR protein levels in HFK cells exposed to 10 mJ/cm^2^ UVB. D. Densitometry of immunoblots (n = 3) and measurements by quantitative RT-PCR of ATR mRNA levels (n = 3) in HPV E6 expressing HFK cells. Levels were normalized to GAPdh (mRNA) or β Actin (protein) for each experiment. Error bars represent standard errors of the mean. * denotes a statistically significant difference from LXSN cells. E. ATR foci formation was measured using immunofluorescence following 10 mJ/cm^2^ UVB exposure. Graph shows percentage of cells positive for ATR foci, above background levels.

Having shown the increased persistence of thymine dimers and elevated UVB induced phospho H2AX levels to be dependent on HPV E6 promoting p300 degradation, we wanted to further elucidate the mechanism of attenuated UVB damage repair in HPV 5 and 8 E6 expressing cells. p300 interacts with ATR, a member of the PI3 kinase family, and is necessary for activation of ATR in response to hydroxyurea [47]. In addition to facilitating cell cycle arrest in response to hydroxyurea, ATR also plays a key role in UVB damage repair [41]. Indeed disruption of this signaling by RNAi mediated reduction of ATR levels results in increased sensitivity to UVB exposure much like we have described in HPV E6 expressing cells [55]. Therefore, we hypothesized that the increased UVB sensitivity in β-HPV E6 expressing cells is also the result of reduced ATR levels or activity. To test this hypothesis, we measured ATR protein levels by immunoblot and found the ATR protein levels were significantly reduced in HFK cells expressing HPV 5 and 8 E6 with and without UVB exposure (Figure 5A, B). ATR levels were also reduced to a lesser degree in HT1080 cells expressing HPV 5 and 8 E6(Figure S6) We also measured ATR mRNA levels by quantitative RT-PCR in HPV E6 expressing HFK cells and found significantly lower ATR mRNA levels in HPV 5 (35% reduction), 8 (45% reduction), and 16 E6 (10% reduction) expressing cells (Figure 5B). Interestingly, ATR levels increased following UVB exposure, but this increase was the greatest in HPV 5 and 8 E6 expressing cells (Figure 5A and Figure S7).

Cells expressing HPV Δ8 E6 had thymine dimer persistence and UVB induced DSBs levels that were similar to vector control cells (Figure 3). To determine if these cells also had ATR levels that were similar to vector control cells, we measured ATR protein and mRNA levels in HPV Δ8 E6 expressing HFK cells. Consistent with the hypothesis that reduced ATR levels in HPV 5 and 8 E6 expressing cells results in more persistent thymine dimers and a greater number of UVB induced DSBs, ATR protein and mRNA levels in HPV Δ8 E6 expressing cells are similar to control cells (Figure 5C and 5D).

In response to UVB exposure, ATR forms distinct nuclear foci, where it co-localizes with and phosphorylates other DNA repair factors [41], but p300 is required for ATR activation [47]. We therefore hypothesized that HPV 5 and 8 E6 expression, by promoting degradation of p300, would interfere with ATR activation in response to UVB exposure. To test this hypothesis, we measured the formation of ATR foci by immunofluorescence following exposure to 10 mJ/cm^2^ UVB exposure. ATR levels peaked in both vector control and HPV 38 E6 expressing HT1080 cells within one hour of exposure to UVB (Figure 5E and Figure S7). In contrast, ATR foci formation does not peak in HPV 5, 8, and 16 E6 expressing cells until 4–6 hours after UVB exposure (Figure 5E and Figure S8). Despite the temporal differences in ATR activation, HPV 5, 8 and 16 E6 expressing cells, at peak, had approximately the same number of ATR positive cells as the vector control cells. Finally, HPV 16 E6 had a significantly delayed ATR peak compared to vector control cells, despite having elevated ATR levels.

**Figure 6:**
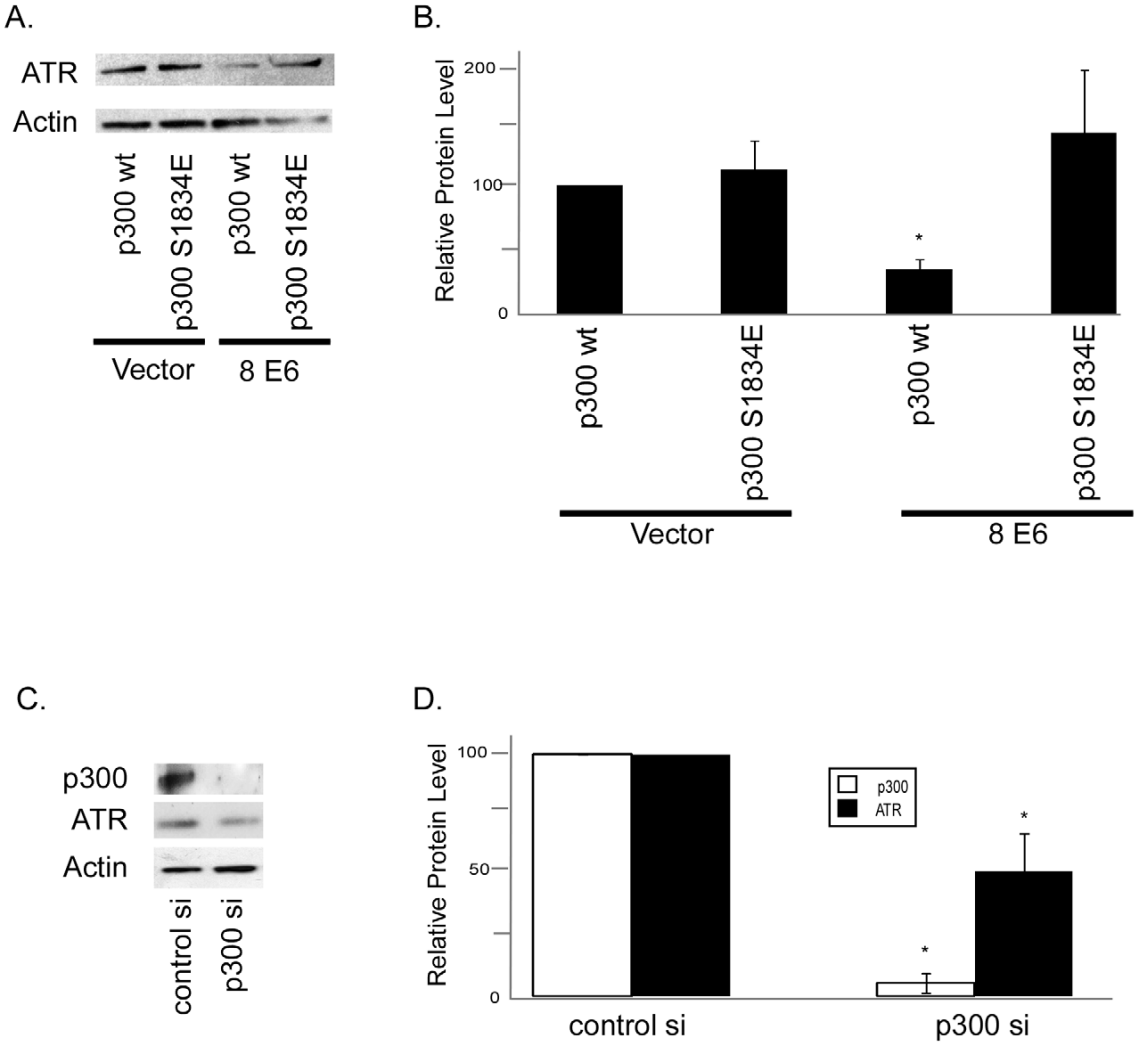
Reduced ATR protein levels in HPV 5 and 8 E6 expressing cells is dependent on p300 degradation. A. Representative immunoblot showing ATR levels in HFK cells cotransfected with either p300wt or p300 S1834E and vector control or HPV 8 E6. β Actin was used as a loading control. B. Densitometry of immunoblots (n = 3) of ATR protein levels in HPV E6 expressing HFK cells cotransfected with either p300wt or p300 S1834E and vector control or HPV 8 E6. Levels were normalized to β actin for each experiment. Error bars represent standard errors of the mean. * denotes a statistically significant difference from HFK cells cotransfected with vector control and p300wt. C. p300 was knocked down in HFK cells by transfection with a pool of 4 siRNAs targeting p300 or a pool of 4 non-targeting siRNAs. Representative immunoblot showing ATR and p300 levels in these cells HFK cells 72 hours post transfection. β Actin was used as a loading control. D. Densitometry of immunoblots (n = 3) of ATR and p300 protein levels in HFK cells transfected with a pool of 4 p300 targeting siRNAs or a pool of 4 non-targeting siRNAs. Levels were normalized to β actin for each experiment. Error bars represent standard errors of the mean. * denotes a statistically significant difference from HFK cells transfected with control siRNAs.

To more definitively test if the HPV E6 mediated reduction in p300 resulted in the reduction of ATR expression, we cotransfected either HPV 8 E6 or a vector control with either a wild-type p300 or a phosphomimetic mutant (p300 S1834E) previously shown to resist HPV 8 E6 mediated degradation [36]. We then measured steady state ATR protein levels by immunoblot. ATR levels were unchanged by the cotransfection of wild type or mutant p300 with a vector control (Figure 6A and B). As expected, ATR levels were reduced in HFK cells cotransfected with wild type p300 and HPV 8 E6. Importantly, ATR levels were similar to control levels in HFK cells cotransfected with HPV 8 E6 and the phosphomimetic p300 mutant (Figure 6A and B). Finally, to provide further support of the hypothesis that HPV 5 and 8 E6 expression reduces ATR levels, we target p300 for siRNA mediated knocked down and show a corresponding reduction in ATR levels (Figure 6C and D).

### Delayed UVB induced p53 accumulation in β HPV E6 expressing cells

**Figure 7:**
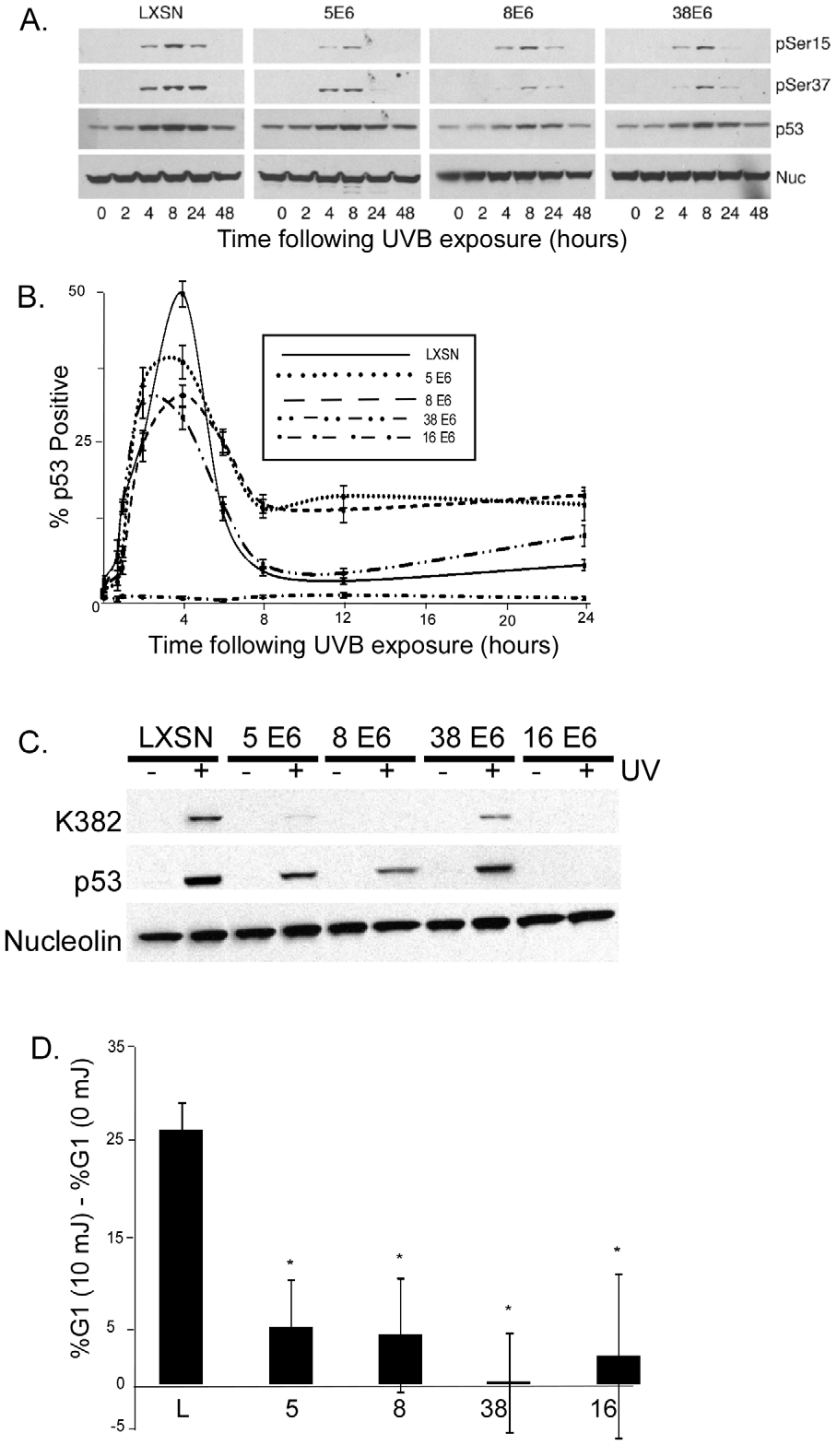
UVB induced p53 response is attenuated in HPV E6 expressing cells. A. Representative immunoblot showing total p53 and phosphorylated p53 (Ser15 and Ser37) levels in β-HPV E6 expressing HFKs following 10 mJ/cm^2^ UVB exposure. Nucleolin levels were used as a loading control. B. p53 levels were measured by immunofluorescence following 10 mJ/cm^2^ UVB exposure in HPV E6 expressing HFKs following 10 mJ/cm^2^ UVB exposure. Graph shows percentage of cells with p53 levels above background. n≥5, and error bars represent standard errors of the mean. In this experiment, HPV 16 E6 served as a negative control because of its ability to degrade p53. C. Representative immunoblot showing total p53 and acetylated p53 (Lys382) levels in HPV E6 expressing HFK either 24 hours after exposure to 10 mJ/cm^2^ UVB or mock exposure. D. Following density arrest in G1, HT1080 cells were either exposed to 10 mJ/cm^2^ UVB or left untreated. This graph shows the difference between the percentage of cells in G1 in UVB exposed cells compared to untreated cells 24 hours after UVB exposure. n≥3. Error bars represent standard errors of the mean. * denotes a statistically significant difference from LXSN cells.

Typically in response to UVB exposure, ATR, a member of the PI3 kinase family, phosphorylates p53 at ser 15 and 37 [41]. This phosphorylation blocks the MDM2 mediated ubiquitination of p53 that in turn leads to its proteasomal degradation. Thus ATR dependent phosphorylation of p53, in response to UVB exposure leads to the accumulation of p53 and activation of p53 responsive genes responsible for DDR [40]–[43], [45], [46]. Having seen a reduction in ATR activation, as well as mRNA and protein levels in HPV 5 and 8 E6 expressing cells, we hypothesized that UVB-induced ATR phosphorylation of p53 may also be attenuated in these cells. To test this hypothesis, we measured levels of phosphorylated p53 (Serine 15 and 37) in HPV E6 expressing cells by immunoblot. HPV 5, 8, and 38 E6 expression in HFK and HT1080 cells was able to significantly reduce phosphorylation of p53 at both Serine 15 and Serine 37 (Figure 7A, and data not shown). Because phosphorylation of p53 by ATR results in p53 accumulation, we measured total p53 levels by immunofluorescence and found that while the majority of vector control cells reach peak p53 levels 8 hours following UV exposure, most HPV 5, 8, and 38 E6 expressing cells do not reach peak p53 levels until 12 hours after the same exposure (Figure 7B and Figure S9). Importantly, p53 accumulation in Δ8 E6 expressing HFK cells closely resembles the p53 accumulation measure in control cells following UVB exposure (Figure S10).

In addition to being phosphorylated in response to UVB exposure, p53 is also acetylated by p300 at lysine 382 [56]. This acetylation has been shown to be important for regulation of cell cycle arrest by p53. Because HPV 5 and 8 E6 promote p300 degradation we hypothesized that p53 acetylation in response to UVB exposure would be reduced in these cells. To test this hypothesis, we measured p53 acetylation by immunoblot in response to UVB exposure in HPV 5, 8, 38, and 16 E6 expressing HFK cells. As predicted, p53 acetylation levels were greatly attenuated in HPV 5 and 8 E6 expressing cells (Figure 7C). p53 acetylation levels were modestly reduced in HPV 38 E6 expressing cells and total p53 levels were greatly reduced in HPV 16 E6 expressing cells.

As a result of p53 accumulation in response to DNA damage, the cell cycle is halted in order to provide time to repair damage DNA [57]. HPV depends on host replication machinery to replicate its genome; therefore it is beneficial for the virus to keep cells cycling by avoiding such delays. While β-HPV E6 is unable to target p53 for degradation like HR α-HPV E6, we hypothesized that by abrogating ATR expression and foci formation, β-HPV E6 expression is able to attenuate UV damage induced G1 arrest [57]. To test this hypothesis, we synchronized HT1080 cells in G1 by density arrest, exposed them to UVB and then compared their ability to recover from this arrest to similarly arrested, but not UV treated cells. As expected, UVB exposure increased the number of control cells in G1 by approximately 30% compared to untreated cells. However, there was no significant difference G1 content in UVB exposed or untreated HPV 5, 8, 38, and 16 E6 expressing cells (Figure 7D).

We have shown that HPV 5 and 8 E6 expression reduces ATR expression, in a manner dependent on p300 degradation, and that following UVB exposure p53 phosphorylation and accumulation as well as cell cycle arrest are attenuated correlating with an increased prevalence of UVB induced DSBs. In order to provide additional evidence that HPV E6 mediated reduction in ATR expression and activity accounted for the increased sensitivity to UVB exposure, we measured the ability of PI3 kinase inhibitors to sensitize cells to UVB exposure. If HPV E6 mediated reduction of ATR results in sensitivity to UVB exposure, then the ability of chemical inhibition of ATR activity to further increase this sensitivity should be less in HPV E6 expressing cells. Currently, ATR activity can only be chemically inhibited by broad spectrum inhibitors, like wortmannin and caffeine that target the multiple PI3 kinase family members, a group of DNA damage responsive kinases, including ATM, DNA-PK, as well as ATR.

**Figure 8:**
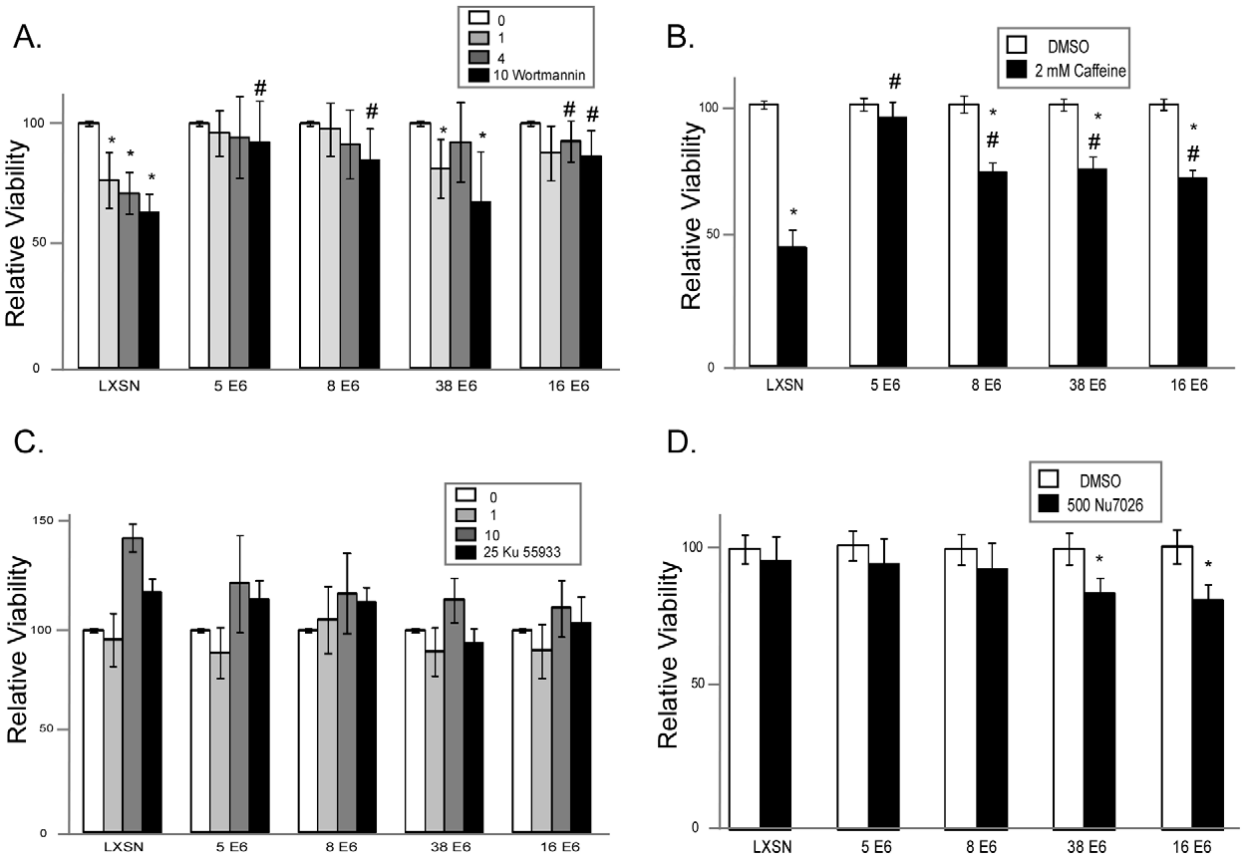
HPV E6 expression reduces sensitivity to combined ATR inhibition and UVB exposure. A. Relative viability of HFK cells expressing HPV 5, 8, 38, and 16 E6 was measured following exposure to 10 mJ/cm^2^ UVB in the presence of a gradient of Wortmannin concentrations (µM). Data was normalized to cells exposed to UVB in the absence of Wortmannin for each cell line. n≥3. Error bars represent standard errors of the mean. B. Relative viability of HFK cells expressing HPV 5, 8, 38, and 16 E6 was measured following exposure to 10 mJ/cm^2^ UVB in the presence or absence of Caffeine (2 mM). Data was normalized to cells exposed to UVB in the absence of Wortmannin for each cell line. n≥3. Error bars represent standard errors of the mean. C. Relative viability of HFK cells expressing HPV 5, 8, 38, and 16 E6 was measured following exposure to 10 mJ/cm^2^ UVB in the presence of a gradient of Ku 55933 concentrations (nM). Data was normalized to cells exposed to UVB in the absence of Ku 55933 for each cell line. n≥3. Error bars represent standard errors of the mean. D. Relative viability of HFK cells expressing HPV 5, 8, 38, and 16 E6 was measured following exposure to 10 mJ/cm^2^ UVB with or without Nu 7026 (nM). Data was normalized to cells exposed to UVB in the absence of Nu 7026 for each cell line. n≥3. Error bars represent standard errors of the mean. * denotes a statistically significant difference between cells exposed with and without inhibitor. # denotes a statistically significant difference from LXSN cells for the same concentration of inhibitor.

Wortmannin is capable of inhibiting ATM and DNA-PK activity at lower concentrations and inhibiting ATM, DNA-PK, and ATR at a concentration of 10 µM [58]. To determine if the increased sensitivity of HPV E6 expressing cells is associated with the reduction of ATR activity in these cells, we measured the ability of a gradient of wortmannin to sensitize HPV 5, 8, 38, and 16 expressing cells to UVB exposure. Importantly, HPV 5, 8, and 38 E6 cells were significantly less sensitive to 10 µM wortmannin in combination with UVB exposure than vector control cells (Figure 8A). Similarly, exposure to 2 mM caffeine inhibits both ATM and ATR activity [59]. To confirm that increased sensitivity of HPV E6 is associated with diminished ATR activity, we measured the ability of caffeine to sensitize cells to UVB exposure. As expected, vector control cells were more sensitive to caffeine combined with UVB exposure than HPV E6 expressing cells (Figure 8B).

Since wortmannin is able to inhibit not only ATR but also ATM, and DNA-PK, and because unlike ATR, chemical inhibitors specific to ATM and DNA-PK are commercially available, we used these more specific inhibitors to confirm that ATR inhibition and not ATM or DNA-PK was responsible for the changes to UVB sensitivity. First, we showed that ATM inhibition by Ku 55933 is unable to sensitize cells to UVB exposure (Figure 8C). We next tested the ability of DNA-PK inhibition by NU7026 to sensitize cells to UVB exposure. Interestingly, DNA-PK inhibition significantly increased UVB sensitivity in HPV 38 and 16 E6 expressing cells, however sensitivity was not increased in either HPV 5 or 8 E6 expressing cells compared with control cells (Figure 8D).

## Discussion

We show that β HPV 5 and 8 E6 decrease at both the mRNA and protein levels of ATR, a PI3 kinase family member that plays a key role in the repair of UVB DNA damage (Figure 5). Additionally, UVB induced ATR foci formation is also delayed in β HPV 5 and 8 E6 expressing cells (Figure 5E). We confirm an earlier observation that thymine dimers persist longer in HPV 5 E6 expressing cells and show that this is also true for HPV 8 and 16 E6 expressing cells following UVB exposure (Figure 1A, 1B, and Figure S2). Importantly, we show that ATR protein levels remain at control levels in cells expressing HPV Δ8 E6, a mutant that has lost the ability to promote p300 degradation. Additionally, when p300 levels are reduced by targeted siRNA, ATR levels are also diminished. Conversely, when HPV 8 E6 and a mutated p300 resistant to HPV 8 E6 mediated degradation were cotransfected ATR levels were not attenuated. Cells expressing HPV Δ8 E6 have significantly less persistent thymine dimers following UVB exposure. We see increased UVB induced DSBs in HPV 5, 8, and 16 E6 expressing cells; rarely in HPV 38 E6 expressing cells; but not in LXSN or HPV Δ8 E6 expressing cells. UVB induced DSBs can be caused by the collapse of a replication fork following collision with a thymine dimer. The increase in UVB induced DSB in HPV 5, 8, and 16 E6 expressing cells with increased thymine dimer persistence and decreased UVB induced G1 arrest (Figure 1 and 7D), is consistent with this explanation of UVB induced DSB formation. H2AX is phosphorylated in an ATR dependent fashion following replication stress, so it unlikely that these elevated H2AX levels result from increased phosphorylation of H2AX in the absence of an increased number of DSBs [60]. In addition to disrupting UVB DNA damage repair, we demonstrate that the HPV 5 and 8 E6 expression sensitizes cells to UVB exposure. Finally, using wortmannin and caffeine, chemical inhibitors of PI3 kinase activity, we demonstrate that this sensitivity is due to the reduced ATR expression in HPV 5 and 8 E6 expressing cells (Figure 8). These data support the hypothesis that β-HPV infections can contribute to NMSC by acting as a co-factor that increases the mutagenic potential of UVB exposure.

Our work shows that β-HPV 5 and 8 E6 expression is able to functionally inhibit UVB DNA damage repair by promoting p300 degradation [36] resulting in decreased ATR activity. As expected by the central role of ATR in UVB induced DNA damage repair, cells expressing β-HPV E6s that reduce ATR levels are more sensitive to UVB exposure. We can mimic this increased UVB sensitivity in control cells by the Inhibition of ATR activity by wortmannin. Although wortmannin treatment increases UVB sensitivity in HPV 5 and 8 E6 expressing cells, the increase in UVB sensitivity is half that seen in control cells (Figure 8). This observation allows us to estimate that HPV 5 and 8 E6 expression reduces the ability of ATR to protect cells against UVB induced damage by approximately 50%.

Attenuated phosphorylation and delayed accumulation of p53 offers further evidence that the HPV E6 mediated reduction of ATR levels and delay in ATR foci formation is physiologically relevant (Figure 5, 7A and 7B). p53 plays a central role in DNA Damage Repair (DDR). In response to UVB induced DNA damage, phosphorylation of p53 blocks its MDM2 mediated degradation allowing p53 to accumulate in the nucleus and activate expression of genes involved in DDR. While β-HPV E6 expression is not able to target p53 for proteasomal degradation like HR α- HPV E6, β-HPV E6 expression still hinders p53 activity. As a result of reduced ATR and p300 levels, p53 phosphorylation and acetylation is diminished in response to UVB induced DNA damage. Indeed, HPV 5 and 8 E6 expression results in the attenuation of p53 accumulation. This provides an explanation for the observation that the repair of thymine dimers is delayed with β-HPV E6 expression. Together these data show that p300 plays an important role in modifying p53 in response to UV damage, not only directly, but also indirectly through ATR.

In addition to its role in DDR, in order to allow time for repair before entrance into the cell cycle, p53 signaling can induce cell cycle arrest. In response to UVB exposure, cells arrest in the G1 phase of the cell cycle. β-HPVs do not encode their own replication machinery and therefore require cycling cells in order to proliferate. We show that HPV 5, 8, 38, and 16 E6 expression allows cells to continue through the cell cycle after UVB exposure in much the same way as unexposed cells (Figure 6D).

Importantly, we show that although reduced UVB induced cell cycle arrest is likely advantageous for β-HPVs, in combination with delayed thymine dimer repair it can be very deleterious for cells. Since β-HPV E6 expressing cells are less likely to experience growth arrest and more likely to contain unrepaired thymine dimers, they are also more likely to have replication forks collide with a thymine dimer resulting in a DSB. Indeed, HPV 5 and 8 E6 expressing cells are nearly 4 times as likely to experience a DSB 8 hours after UV exposure as control cells (Figure 1). The transformation of a thymine dimer into a DSB represents a serious escalation of the damage sustained by the chromosome. Since the repair of a DSB can result in mutations or recombinations, the reduction of ATR activity by β-HPV E6 expression can dramatically alter the persistence as well as the magnitude of damage sustain by UV exposure.

p300 interacts, either directly or indirectly, with ATR and this interaction is necessary for hydroxyurea-induced cell cycle arrest [47]. Indeed, in the absence of catalytically active p300, ATR fails to properly phosphorylate Chk1 [47], one of its downstream targets [61]. Here we show that in cells that have reduced p300 levels due to HPV 5 and 8 E6 expression [36], ATR activation is significantly delayed in response to UVB exposure (Figure 5E). Additionally, we show that ATR mRNA and protein levels are reduced in these cells (Figure 5A and 5B), but not in cells expressing HPV Δ8 E6 (Figure 5C and 5D), suggesting that p300 plays a role in regulating ATR expression. Interestingly, cells expressing HPV 16 E6 also have a delayed ATR response (Figure 5E). However, HPV 16 E6 expressing cells do not have reduced ATR levels (Figure 5A, 5B) and have been shown to bind p300, but not to promote its degradation [36]. We speculate that the delayed ATR response in these cells may be related to the binding of p300 by HPV 16 E6 potentially disrupting the interaction between p300 and ATR necessary for ATR activation.

Interestingly, a reduction in ATR levels as well as diminished ability to repair UVB induced DNA damage (increased thymine dimer persistence, increased DSBs, delayed ATR activation and attenuated p53 accumulation) occurred in HT1080 cells expressing HPV 5 and 8 E6. AKT is constitutively activated in HT1080 cells and this impedes the ability of HPV 5 and 8 E6 to degrade p300 by pushing the equilibrium of AKT/p300/E6 binding toward the stabilizing interaction between AKT and p300. We propose that despite this shift in equilibrium, HPV 5 and 8 E6 would maintain their ability to bind p300, and disrupt some of its downstream interactions, specifically those necessary for ATR activation and expression.

The response to UVB exposure in cells expressing HPV 38 E6 is particularly intriguing. HPV 38 E6 expressing cells share several characteristics with HPV 5 and 8 E6 expressing cells. They fail to G1 arrest in response to UVB exposure and have diminished p53 acetylation and phosphorylation. However, thymine dimer repair persistence and UVB induced DSB formation in HPV 38 E6 expressing cells are more similar to vector control cells. Perhaps these observations are best explained by the ability of HPV 38 E6 to bind p300 in a similar location as by HPV 5 and 8 E6, but with a much lower affinity. We speculate that this weaker interaction gives rise to the phenotypes

While it has been shown by our lab and others that β-HPV E6 expression is capable of promoting Bak degradation following UVB exposure (thus inhibiting apoptosis 24 hours after UV exposure), here we report that expression of the same β-HPV E6s results in decreased proliferation during a three day window following UVB exposure [31], [62]. We speculate that this apparent contradiction can be most readily explained by the temporal differences in the two assays. We describe significantly delayed repair in β-HPV E6 expressing cells and theorize that this may delay apoptosis past the previously observed window. Alternatively, where the previous work looked exclusively at apoptosis, the viability assay described here looks more generally at recovery and growth following UVB exposure. The diminished viability observed using this more inclusive measurement of cellular health may indicate an increase in cellular senescence, delay/reduction in proliferation or an increase in non-apoptotic death.

We speculate that Bak degradation provides an immediate benefit to the virus following UV exposure. β HPV E6 expression drives cells through cell cycle despite the presence of thymine dimers. While this potentially provides the virus access to the cellular transcription machinery, it also increases the likelihood that UV exposure results in a DSB. The ability of β HPV E6 expression to promote Bak degradation at this time may be necessary to allow cells to avoid apoptosis in response to increased levels of DSBs.

If β-HPV infections play a role in NMSC, then they must do so transiently, as testing has rarely been able to show consistent HPV expression in these tumors. The hypothesis most consistent with this evidence is that a β-HPV infection acts in a way that initiates tumor development, but that is not necessary for tumor maintenance. Our data are consistent with a model of β-HPV linked skin cancer, where a β-HPV infection confers resistance to UV induced apoptosis and cell cycle arrest. Following UV exposure, this resistance then allows more cells to pass through the cell cycle with unrepaired thymine dimers and thereby increasing the prevalence of DSBs. While this would result in reduced cellular viability, the surviving population of cells would be at risk for increased mutations, particularly those that allow cells to tolerate genomic instability and thus increase their carcinogenic potential. Interestingly, several studies have found p53 mutations to be prevalent in skin malignancies from patients with epidermodysplasia verruciformis (EV), a rare genetic disorder associated with increased susceptibility to HPV 5 and 8 infections [7], [63], [64]. Finally, in support of a model where HPV 5 and 8 infections promote tumorigenesis by inhibiting UV damage repair, a high frequency (50–75%) of these mutations are C→T transitions, typically result of UV damaged DNA [63], [64].

## Materials and Methods

### UVB irradiation

Cells were seeded and grown overnight. The following morning, cells were washed once with PBS and then irradiated through a thin film of PBS with the amount of UVB irradiation indicated in the figure. Fresh media was then added to the cells. The UVB source is a parallel bank of two FS20t12/UVB bulbs (Solarc Systems, Inc., Barrie, ON, Canada) with an output range of 280 to 320 nm. The UVB output is measured with an IL1400A radiometer coupled with the SEL240/UVB-1/TD UVB detector (International Light, Peabody, MA).

### Cellular viability assay

10,000 cells per well were seeded in a 24 well plate. 12 hours later, the cells were either exposure to UVB or switched to media containing either cisplatin (Sigma-Aldrich, St. Louis, MO) or mitomycin C (Acros Organics, Geel, Belgium). After three days, cells were stained with 0.2% (w/v) crystal violet in 5% (v/v) acetic acid and 2.5% (v/v) isopropanol for one hour. The plates were then washed with water and dried. Crystal violet stain was then eluted from the cells by one wash in 10% (v/v) acetic acid and the amount of stain was quantitated by measuring absorbance at 590 nanometers using KC Junior software and a μQuant spectrophotometer (Bio-tek Instruments, Winooski, VT) as a measure of the number of cells. The linear range for crystal violet absorption and its ability to accurately quantitate cells was verified (Figure S11).

### DNA constructs

All HPV E6 constructs were cloned into the pLXSN vector after being generated by PCR amplification from full length HPV DNA. The construction of these vectors has been previously described [31]. p300wt and p300S1834E vectors have been previously described [36].

### RT-PCR

RNA was isolated with Trizol reagent (Invitrogen, Carlsbad, CA). Briefly, 1 ml of Trizol was added to each 10-cm plate, cells were incubated for 5 minutes, and 200 µL of chloroform was added. The aqueous phase was transferred and mixed with an equal volume of isopropanol, incubated for 10 minutes, and pelleted at 12,000 rpm for 10 minutes at 4°C. After being washed with 75% ethanol, the samples were again pelleted at 9,000 rpm for 5 minutes and resuspended in in RNase free water. 1 µg of total RNA was reverse transcribed to generate cDNA, using the iScript cDNA synthesis kit (BioRad, Hercules, CA). As a negative control, parallel samples were run without reverse transcriptase. Non-quantitative PCR amplification was then performed to identify 100 bp amplicons with E6 and 36B4 primers as previously described [31]. For real-time RT-PCR, RNA was isolated and reverse transcribed as above and quantitative real-time PCR was performed using an ABI 9700 sequence detection system (Applied Biosystems, Foster City, CA). Amplification was carried out using TaqMan primer/probes: GAPDH (4333764F) and ATR (Hs00354807) according to the manufacturer's instructions (Applied Biosystems, Foster City, CA). Reactions were performed in triplicate in a 25 µL volume, with the following cycle parameters: enzyme activation (10 minutes at 95°C), followed by 40 cycles (each cycle consisting of 15 seconds at 95°C and 1 min at 60°C). Data analysis was performed using the comparative threshold cycle method (Applied Biosystems, Foster City, CA) to determine expression levels.

### Immunofluorescent microscopy

8,000 HFKs or HT1080 cells (vector control, HPV 5 E6, 8 E6, 38 E6 and 16 E6) were seeded onto Greiner glass bottomed 96well dishes. The next day, at the appropriate time following UV exposure, cells were fixed for 15 min with 4% paraformaldehyde at 4C while spinning at 200RPM in tabletop centrifuge and permeabilized with ice cold Methanol/Acetone (1∶1) for 5 min at 4C. Cells were washed carefully with PBS 3X then blocked with PBS/10% Goat serum for 30 min at 37C, Washed 2× PBS and then incubated with primary antibody to phospho-H2AX (ser139) (Upstate-Millipore, Billerica, MA), p53 (Cell Signaling, Danvers, MA), or thymine dimmers(Cosmo Bio, Tokyo, Japan). The cells were then washed and incubated with the appropriate secondary antibodies (Alexa Fluor 488 anti mouse IgG (Invitrogen, Carlsbad, CA) 1∶1000+ Hoechst 33342 DNA dye 1∶10,000 for 1 hour) before being visualized using the Cellomics Array Scan microscope (Thermo Scientific, Waltham, MA, USA).

### Automated image analysis

Images of the cells stained with the indicated antibodies were acquired with a Cellomics ArrayScan VTI (Thermo Scientific, Waltham, MA, USA) using a 20×0.40 NA objective. Image analysis was performed using Cellomics ArrayScan HCS Reader (Thermo Scienctific), with the XF53 filter set using the compartment analysis profiling module v3.5. Nuclear staining was used to delineate the cells. After background correction, either the nuclear fluorescence intensity (Compartmental Analysis Bioapplication) or the quantity of nuclear foci (Spot Detector Bioapplication) was assessed. Approximately 400 cells were analyzed in each well for all reactions. Each time point and cell line was repeated in triplicate on the plate and in triplicate experimentally.

### Immunoblotting

Whole cell extracts were prepared by mechanically detaching cells in cold PBS and resuspending in WE16th lysis buffer (50 nM Tris-HCL at pH 7.5, 250 mM NaCl, 5 mM EDTA, 1% NP-40, 0.1% sodium dodecul sulfate, 20% glycerol, 80 mM β-glycero-phosphate, 50 mM sodium fluoride, 1 mM sodium orthovanadate, and a COMPLETE protease inhibitor tablet (Roche, Alameda, CA). Lysates were then sonicated and clarified by centrifugation. The DC protein assay (Biorad, Hercules, CA) was used to determine protein concentrations. Equal amounts of protein lysates (15–30 µg) were electrophoresed on SDS-polyacrylamide gels and transferred to Immobilon-P membranes (Millipore, Billerica, MA). These membranes were then exposed to primary antibodies against p53 (Calbiochem, San Diego, CA), ATR (Cell Signaling, Danvers, MA), phospho p53 (ser15 or ser37) (Cell Signaling), nucleolin (Santa Cruz Biotechnology, Santa Cruz, CA), acetyl-p53 (lys382) (cell signaling), or actin (Santa Cruz Biotechnology). After exposure to the corresponding HRP-conjugated secondary antibody, cells were visualized using lumilite HRP substrate (Thermo Fisher Scientific, Rockford, IL).

### Tissue culture

HT1080 cells are a spontaneously immortalized fibrosarcoma that contain a constitutively activate N-Ras gene. Primary human foreskin keratinocytes (HFKs) were derived from neonatal human foreskins and grown in EpiLife medium supplemented with calcium chloride (60 µM), human keratinocyte growth supplement (Cascade Biologics, Portland, OR) and penicillin-streptomycin. Multiple derivations of HFK cells from neonatal human foreskins were utilized in this work. Following viral transduction, and selection, HPV E6 expression was confirmed using rtPCR to measure HPV E6 levels as well as by immunoblot to confirm HPV 5 and 8 E6 expressing cells had previously characterized reduction in p300 [36] and that HPV 16 E6 expressing cells had previously described reduction in p53 [20]. HT1080 and 293T cells were grown in DMEM medium supplemented with 10% Fetal Bovine Serum (FBS).

Briefly HPV E6 genes cloned into a LXSN vector were co-transfected with VSV-G helper plasmids into 293T cells using Fugene 6 (Roche), and retrovirus was collected at 12, 24, 36, and 48 hours post transfection. Transiently produced virus was concentrated by ultracentrifugation and used to infect HFK and HT1080 monolayers (50–60% confluent) in the presence of Polybrene (8 µg/mL). Four hours after infection, cells were washed with PBS and the media replaced. The cells were expanded when confluent and placed under neomycin-G418 selection (50 mg/L) for 7 days. All transient transfections were done using TransIT Keratinocyte transfection reagent (Mirus), and assessed 72 hours later for levels of ATR. siRNA specific for p300 and controls were purchased from Dharmacon RNA Technologies. siRNA transfections were done using RNAiMAX (Invitrogen) according to the manufacturer's guidelines, and were assessed for p300 and ATR levels 72 hours after transfection.

### Nuclei isolation and propidium iodide staining

Cells were harvested by trypsinization and then pelleted by centrifugation at 4°C. The resulting pellets were then resuspended in PBS/2% fetal bovine serum before being pelleted again by centrifugation at 4°C. While vortexing, the pellets were resuspended in 3 mL of cold 95% ethanol. Once thoroughly resuspended, the cells were covered in foil and kept at 4°C overnight. Cells were then pelleted by centrifugation at 4°C and the supernatant was aspirated. To strip the cytoplasm from the cells, the resulting pellets were resuspended with continual vortexing in 0.08% pepsin and incubated at 37°C for 20 minutes. The resulting nuclei were then pelleted by centrifugation at 4°C and the supernatant was removed. The pelleted nuclei were then resuspended with continuous vortexing in 2 mL IFA (150 mM NaCl, 4% fetal bovine serum, 0.1% sodium azide)+5% Tween 20. Nuclei were again pelleted by centrifugation at 4°C and the supernatant was removed. The pellet was resuspended in 250 µL+5 µg/ml RNase A and transferred to 12×75 mm snap cap tubes with filter caps. Following 30 minute incubation at 37°C for 30 minutes 250 µL fo 100 µg/ml propridium iodide (in PBS) was added to each tube. Samples were stored overnight at 4°C before being analyzed for DNA content using BD Bio Canto instrument and FlowJo software.

### Density arrest

Cells were seeded at 50–60% confluence and allowed to grow until they reached 95–100% confluence. Old media was carefully removed and replaced with new media every 24 hours. One day after the cells reached optimal confluence, they were either exposed to 10 mJ/cm^2^ UVB or left untreated as a control, seeded and either allowed to grow for 24 hours to access G1 arrest or harvested immediately to access density arrest.

### PI3 kinase inhibition

All inhibitors were dissolved in DMSO and used at indicated concentration. Caffeine, Wortmannin, and Nu7026 were purchased from Sigma Aldrich (St. Louis, MO). Ku55933 was purchased from Calbiochem (San Diego, CA)

### Statistical analysis

Unless otherwise noted, statistical significance was determined by paired student T test and confirmed when appropriate by two way ANOVA with Bonferroni correction. Only p values less than or equal to 0.05 were reported as significant. Additionally, Microsoft excel was used to provide the curve fitting in Figures 1, 2, 3, 5, and 6. These curves are intended as visual aids and are not as predictive values.

## Supporting Information

Figure S1 **Confirmation of HPV E6 expression.** A. Quantification of HPV E6 expression by qRT-PCR in HFK cells. Levels were normalized to β-globin expression levels. Error bars represent standard errors of the mean. B. Quantification of HPV E6 expression by qRT-PCR in HT1080 cells. Levels were normalized to the expression levels of a house keeping gene, 36B4.
(TIF)

Figure S2 **HPV E6 expression increases thymine dimer persistence following UVB exposure.** Representative fields from HT1080 cells exposed to 10 mJ/cm^2^ UVB with immunofluorescent detection of thymine dimers (green) in DAPI (blue) stained nuclei.
(TIF)

Figure S3 **Increased thymine dimer formation cannot account for HPV E6 induced thymine dimer persistence.** HT1080 cells were exposed to between 0 and 25 mJ/cm^2^ UVB and immunofluorescent detection of thymine dimers was used to quantify the number of thymine dimers generated at each dose. n = 3. Error bars represent standard errors of the mean.
(TIF)

Figure S4 **HPV E6 expression increases H2AX phosphorylation following UVB exposure.** Representative fields from HT1080 cells exposed to 10 mJ/cm^2^ UVB with immunofluorescent detection of phospho H2AX (green) in DAPI (blue) stained nuclei.
(TIF)

Figure S5 **Linear regression analysis of UVB exposed HFK and HT1080 cells.** Relative viability of cells is plotted as a function of UVB exposure. Linear regression lines were calculated by GraphPad Prism software and used to determine the median lethal dose for exposure. A. HFK cells. B. HT1080 cells.
(TIF)

Figure S6 **HPV β E6 expression results in diminished ATR protein levels in HT1080 cells.** Representative immunoblot showing ATR and p300 levels in HT1080 cells. β Actin is used as a loading control.
(TIF)

Figure S7 **ATR protein levels increase in HFK cells following UVB exposure.** Comparison of densitometry of ATR levels measured by immunoblot in HFK cells exposed to 10 mJ/cm^2^ UVB or mock exposed.
(TIF)

Figure S8 **HPV β E6 expression delays ATR activation following UVB exposure.** Representative fields from HT1080 cells exposed to 10 mJ/cm^2^ UVB with immunofluorescent detection of ATR (green) in DAPI (blue) stained nuclei.
(TIF)

Figure S9 **HPV β E6 expression attenuates p53 accumulation following UVB exposure.** Representative fields from HT1080 cells exposed to 10 mJ/cm^2^ UVB with immunofluorescent detection of p53 (pink) in DAPI (blue) stained nuclei.
(TIF)

Figure S10 **Attenuated p53 accumulation in HPV 8 E6 expressing HFK cells is dependent on p300 degradation.** HFK cells were exposed to 10 mJ/cm^2^ UVB and immunofluorescence was used to measure the percentage of cells positive for p53 above background.
(TIF)

Figure S11 **Validation of crystal violet staining accuracy.** A. and B. Crystal violet absorption at 590 nm as a function of crystal violet staining solution shows a limited linear range. C. Crystal violet staining within this linear range accurately reflects colony counts for cells seeded in range from 1,000–25,000/well of a 24 well plate and grown for three days.
(TIF)
