# Supplementary figures and images for: HPV 5 and 8 E6 Abrogate ATR Activity Resulting in Increased Persistence of UVB Induced DNA Damage
Source: PLoS Pathog. 2012 Jul 12;8(7):e1002807. doi: 10.1371/journal.ppat.1002807 (PMC3395675; doi:10.1371/journal.ppat.1002807)

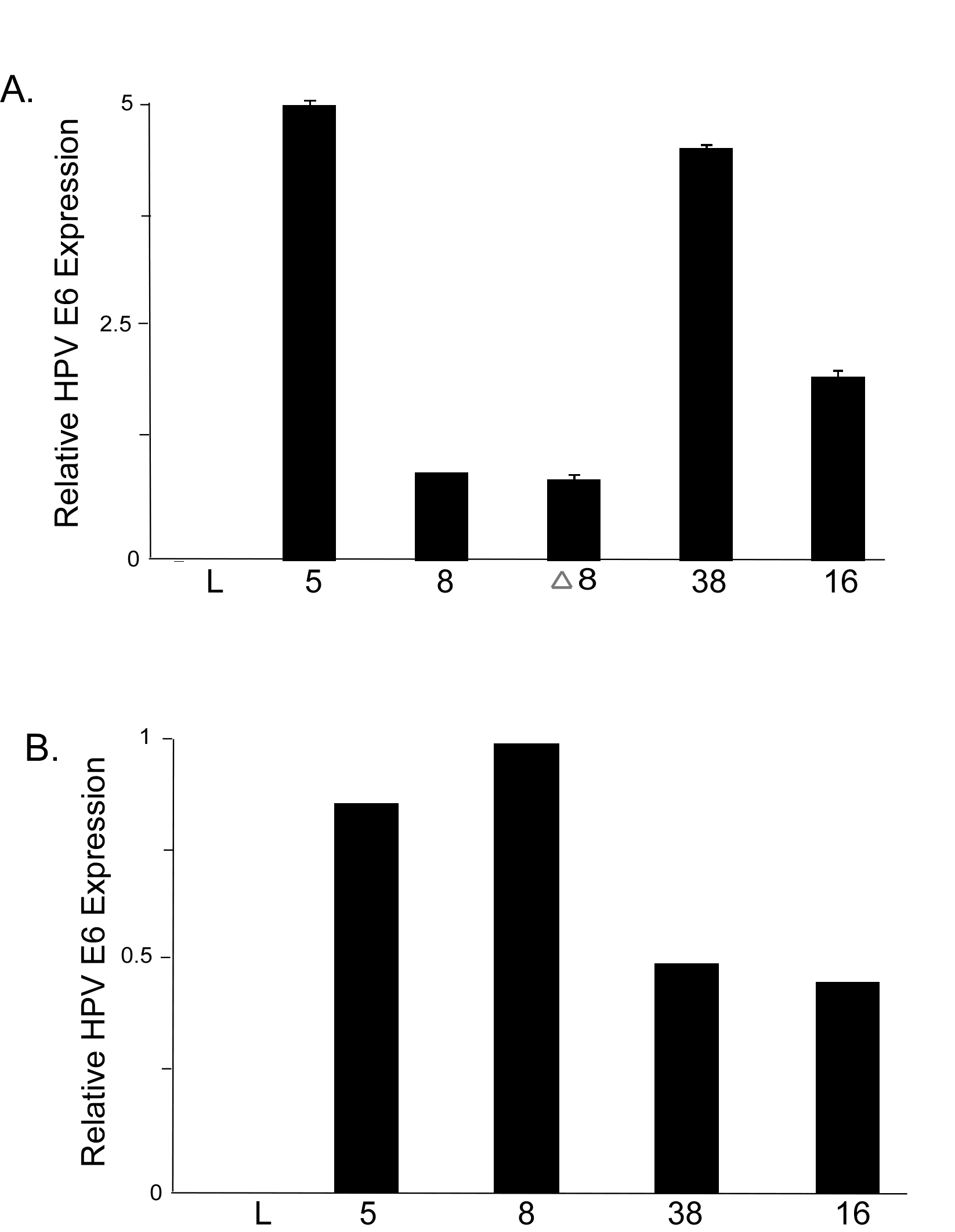

Supplement: Figure S1 — Confirmation of HPV E6 expression. A. Quantification of HPV E6 expression by qRT-PCR in HFK cells. Levels were normalized to β-globin expression levels. Error bars represent standard errors of the mean. B. Quantification of HPV E6 expression by qRT-PCR in HT1080 cells. Levels were normalized to the expression levels of a house keeping gene, 36B4. (TIF) [file ppat.1002807.s001.tif]

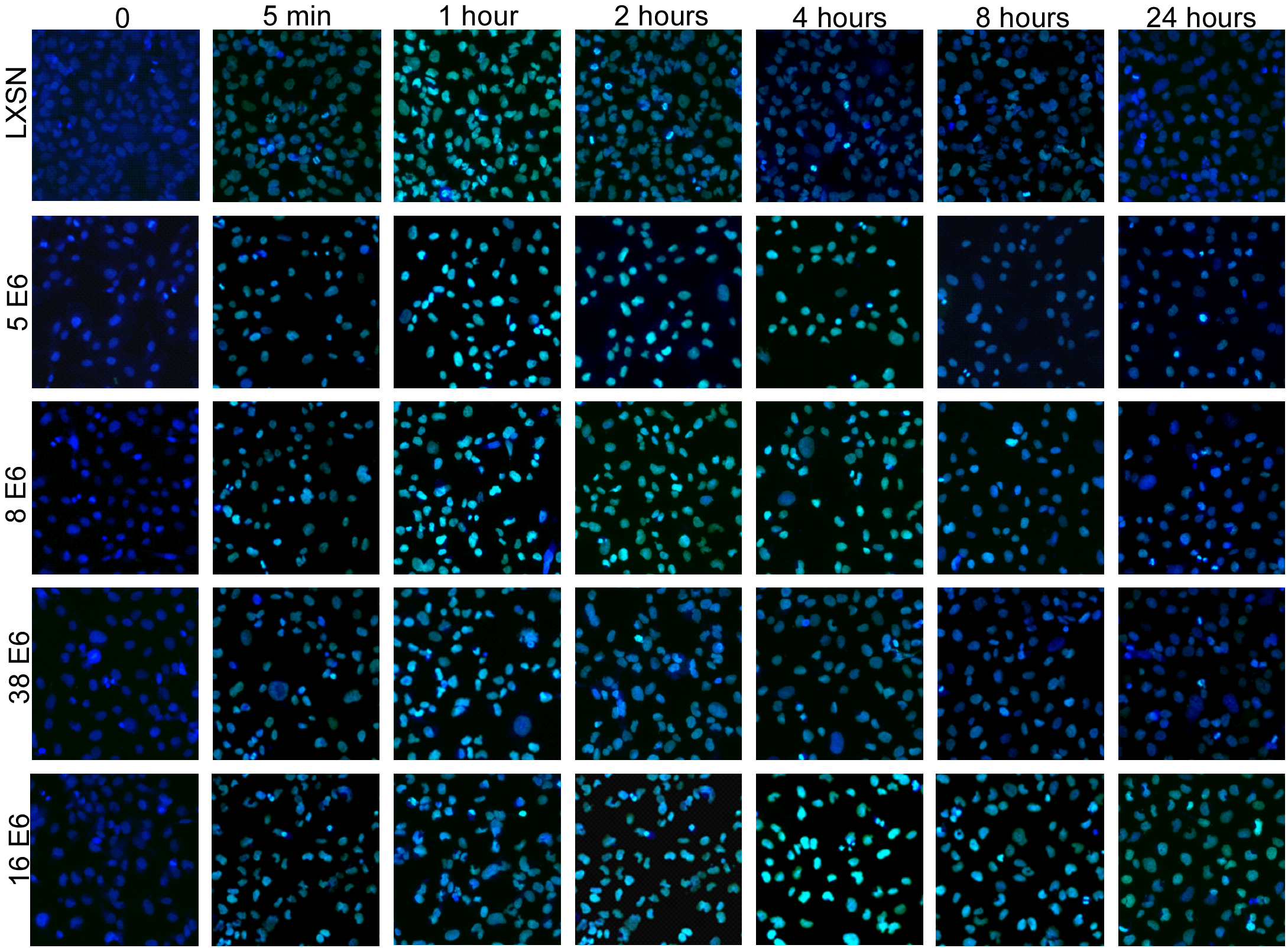

Supplement: Figure S2 — HPV E6 expression increases thymine dimer persistence following UVB exposure. Representative fields from HT1080 cells exposed to 10 mJ/cm2 UVB with immunofluorescent detection of thymine dimers (green) in DAPI (blue) stained nuclei. (TIF) [file ppat.1002807.s002.tif]

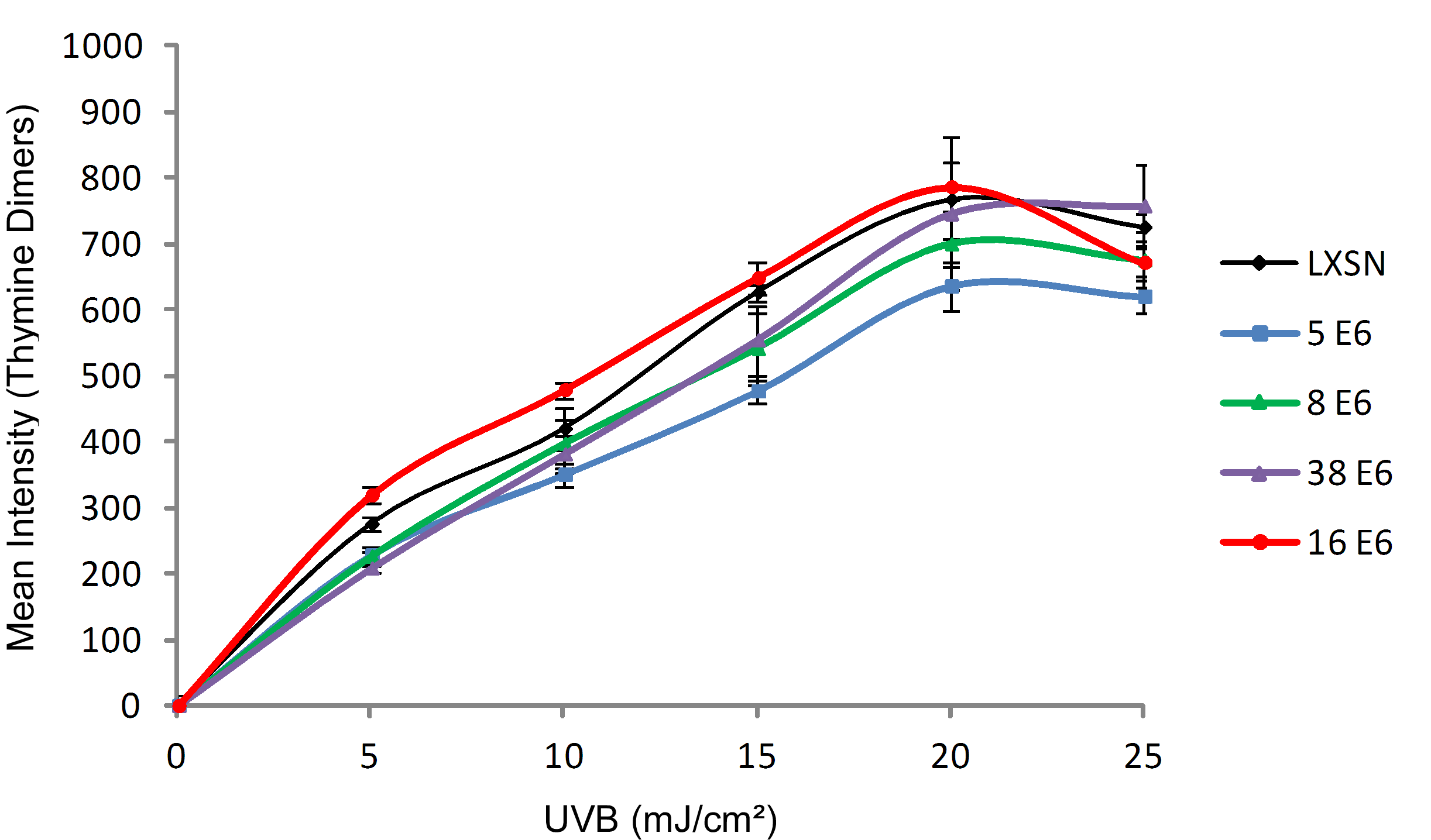

Supplement: Figure S3 — Increased thymine dimer formation cannot account for HPV E6 induced thymine dimer persistence. HT1080 cells were exposed to between 0 and 25 mJ/cm2 UVB and immunofluorescent detection of thymine dimers was used to quantify the number of thymine dimers generated at each dose. n = 3. Error bars represent standard errors of the mean. (TIF) [file ppat.1002807.s003.tif]

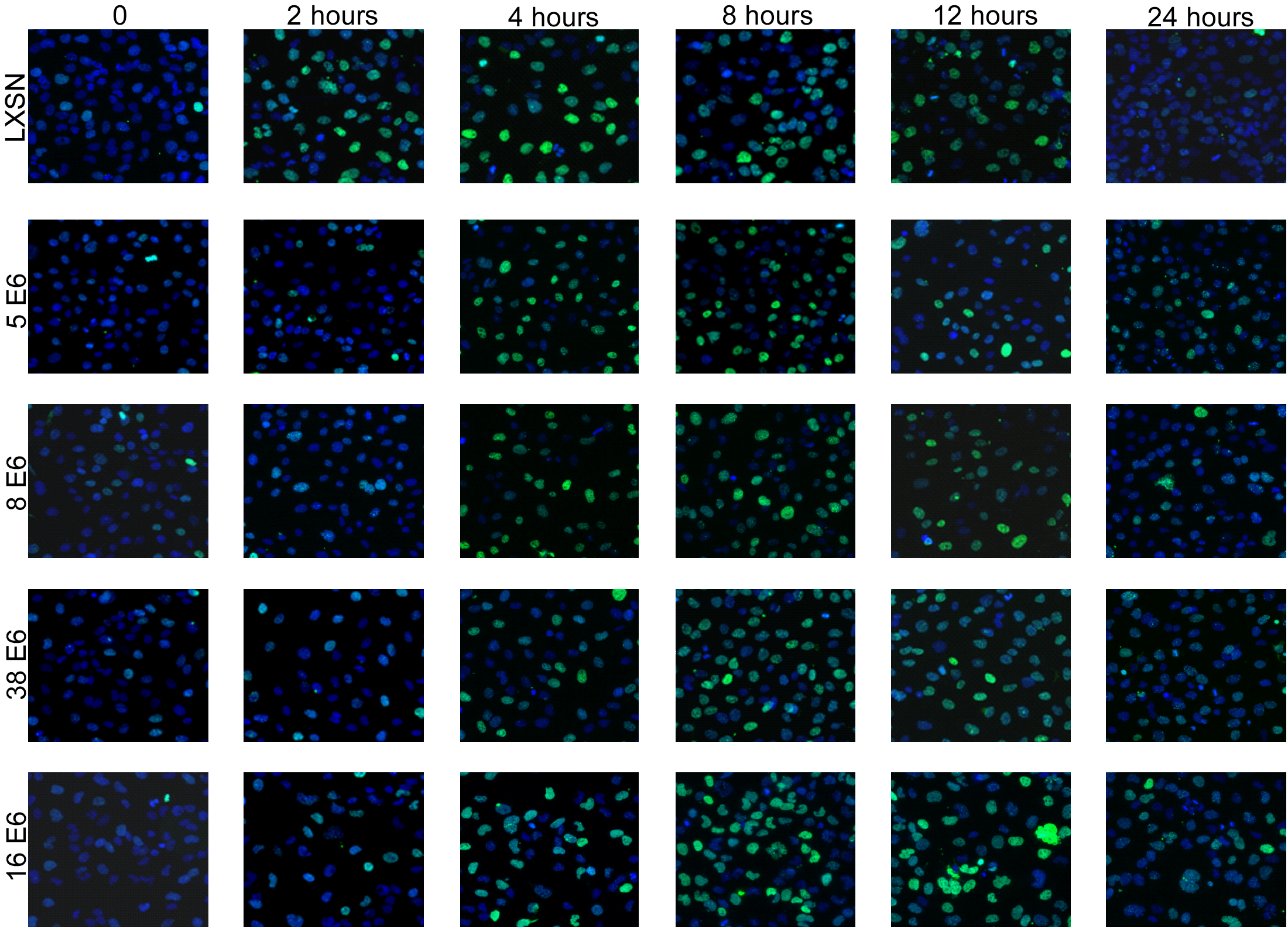

Supplement: Figure S4 — HPV E6 expression increases H2AX phosphorylation following UVB exposure. Representative fields from HT1080 cells exposed to 10 mJ/cm2 UVB with immunofluorescent detection of phospho H2AX (green) in DAPI (blue) stained nuclei. (TIF) [file ppat.1002807.s004.tif]

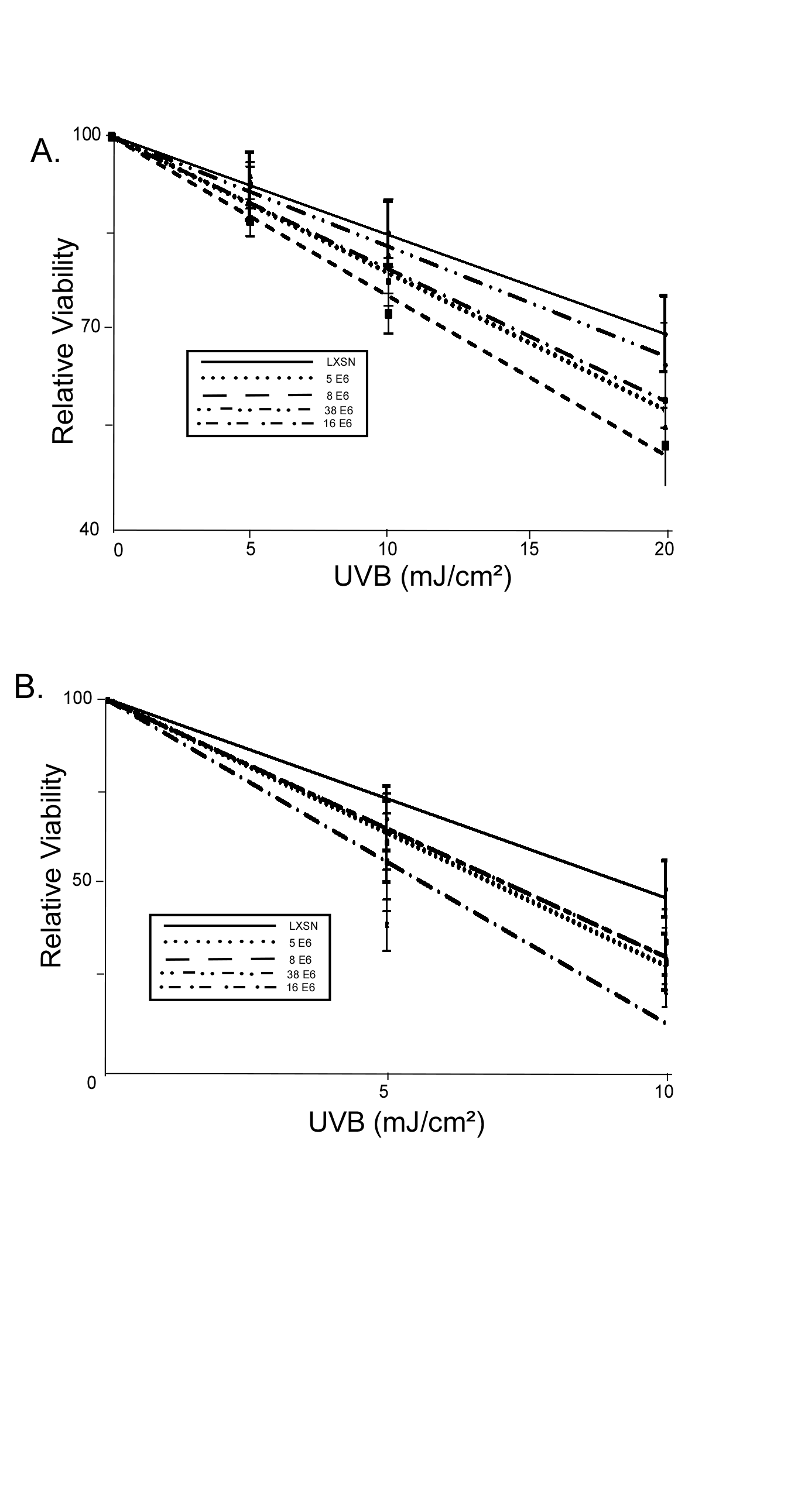

Supplement: Figure S5 — Linear regression analysis of UVB exposed HFK and HT1080 cells. Relative viability of cells is plotted as a function of UVB exposure. Linear regression lines were calculated by GraphPad Prism software and used to determine the median lethal dose for exposure. A. HFK cells. B. HT1080 cells. (TIF) [file ppat.1002807.s005.tif]

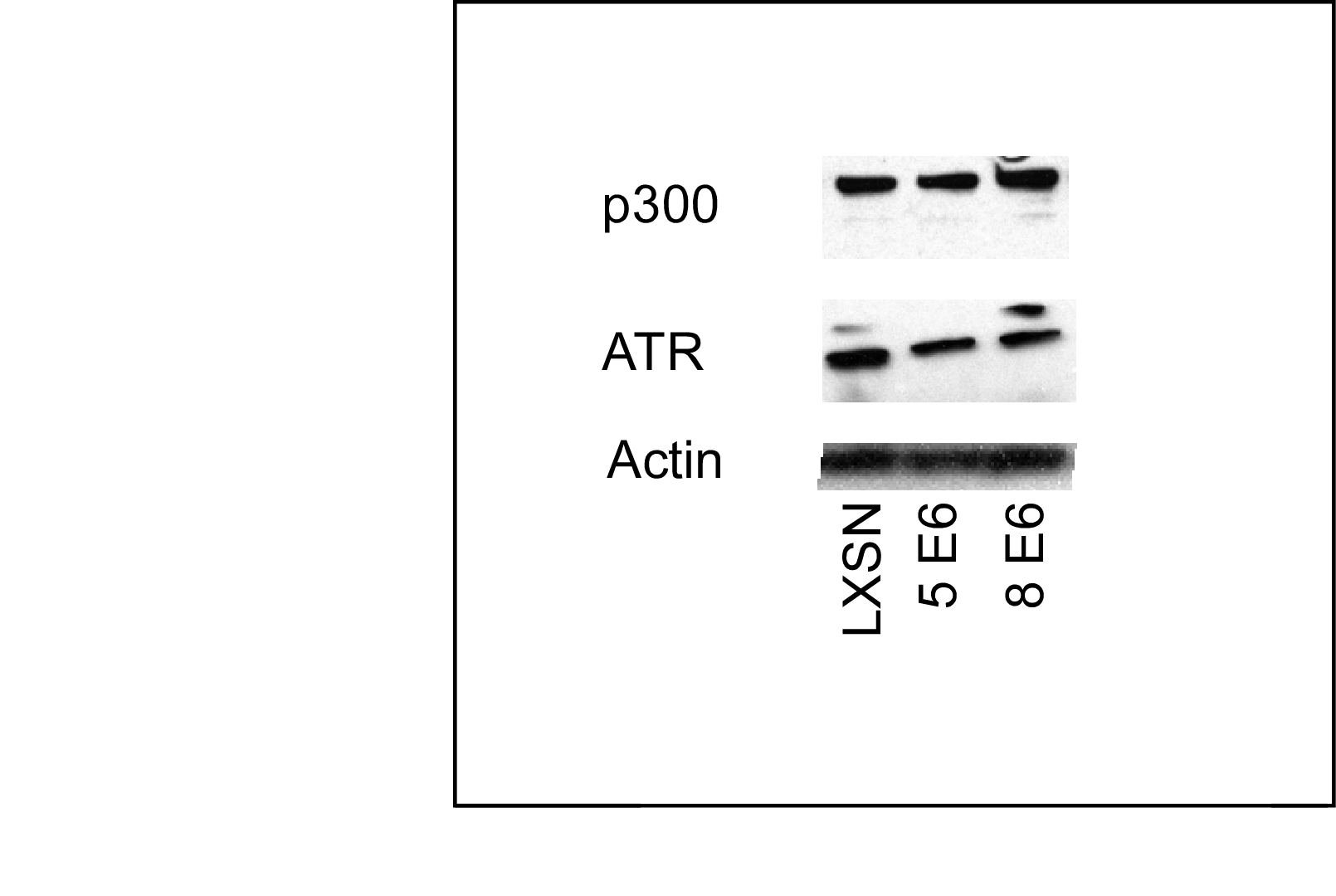

Supplement: Figure S6 — HPV β E6 expression results in diminished ATR protein levels in HT1080 cells. Representative immunoblot showing ATR and p300 levels in HT1080 cells. β Actin is used as a loading control. (TIF) [file ppat.1002807.s006.tif]

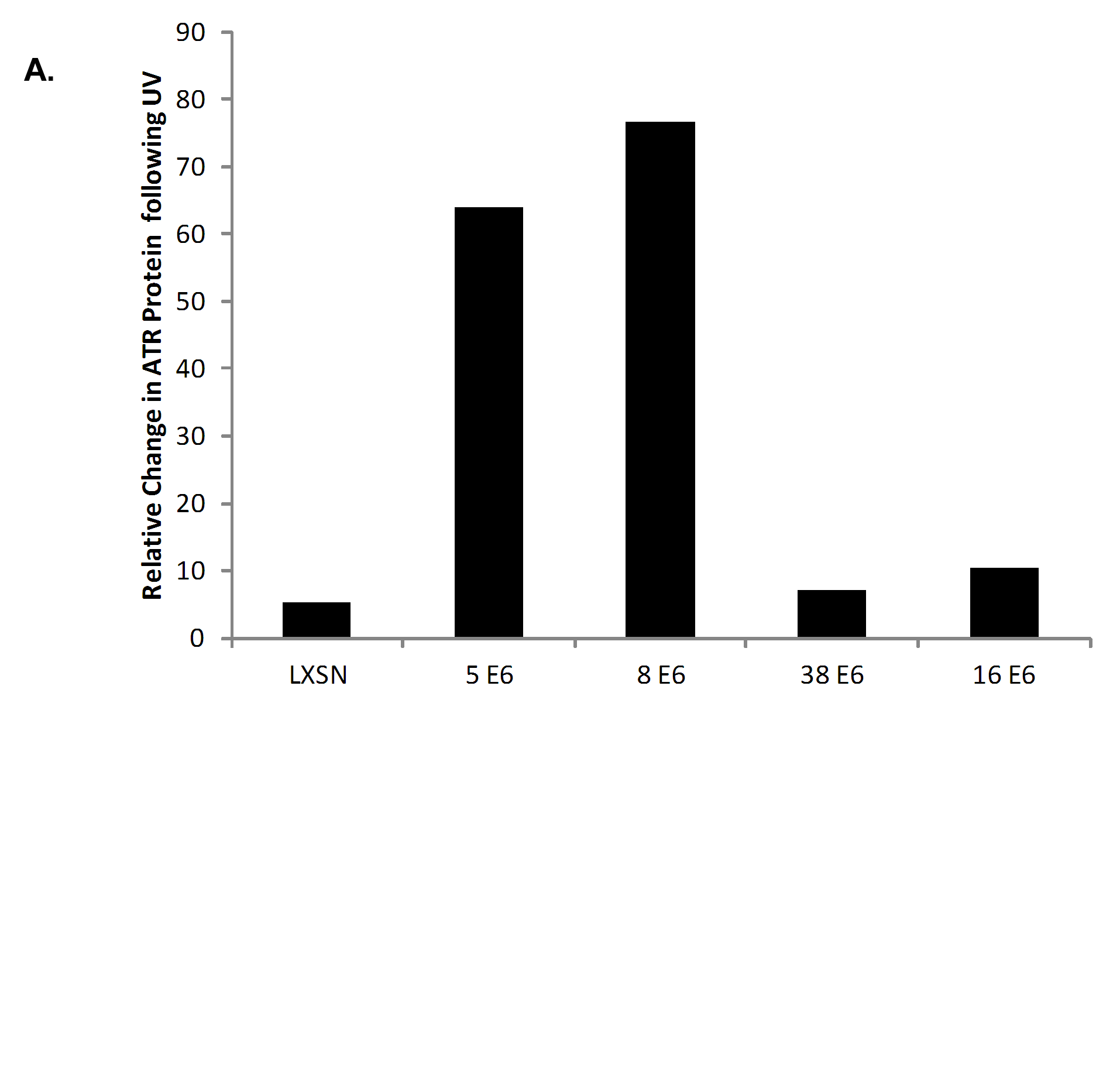

Supplement: Figure S7 — ATR protein levels increase in HFK cells following UVB exposure. Comparison of densitometry of ATR levels measured by immunoblot in HFK cells exposed to 10 mJ/cm2 UVB or mock exposed. (TIF) [file ppat.1002807.s007.tif]

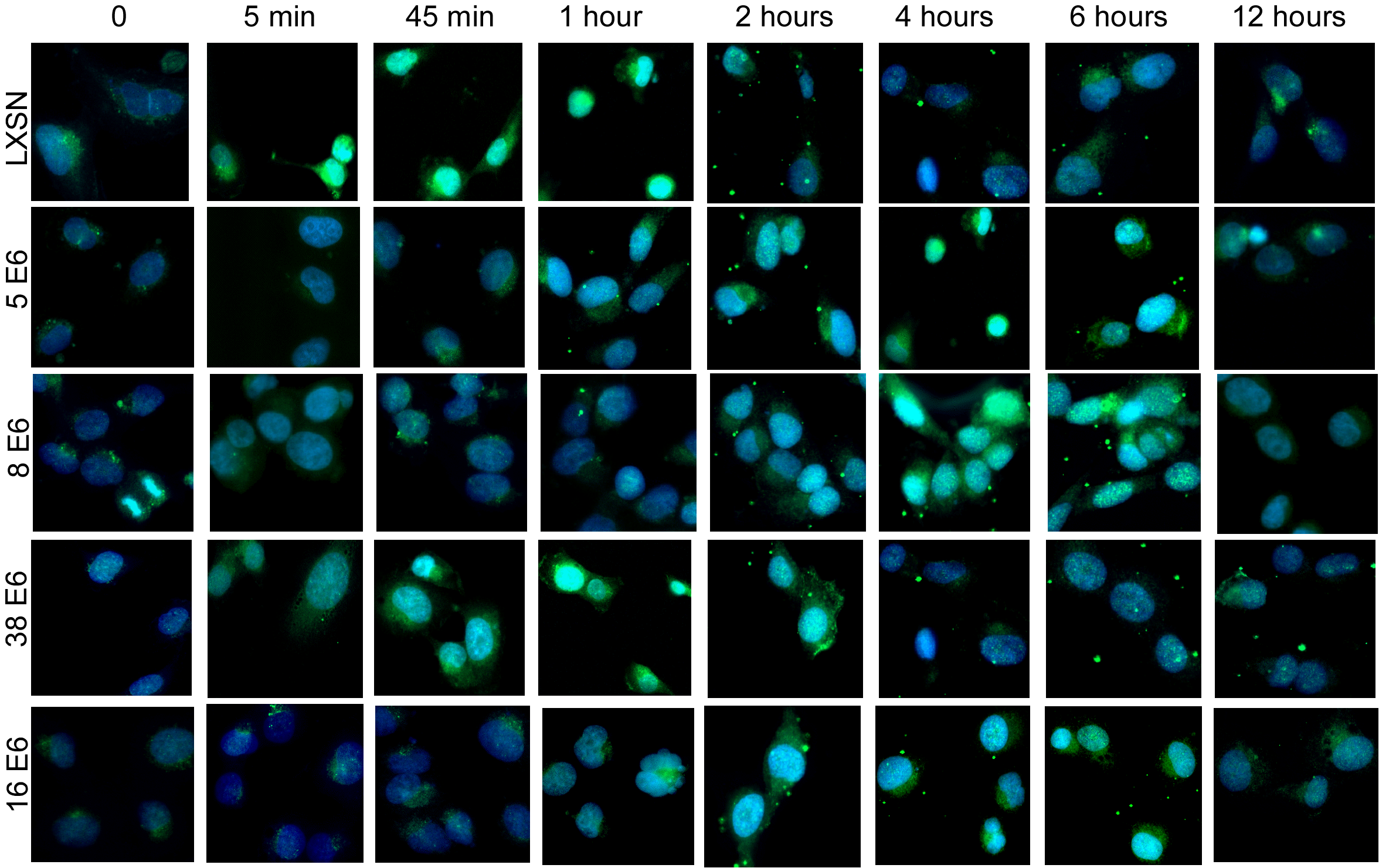

Supplement: Figure S8 — HPV β E6 expression delays ATR activation following UVB exposure. Representative fields from HT1080 cells exposed to 10 mJ/cm2 UVB with immunofluorescent detection of ATR (green) in DAPI (blue) stained nuclei. (TIF) [file ppat.1002807.s008.tif]

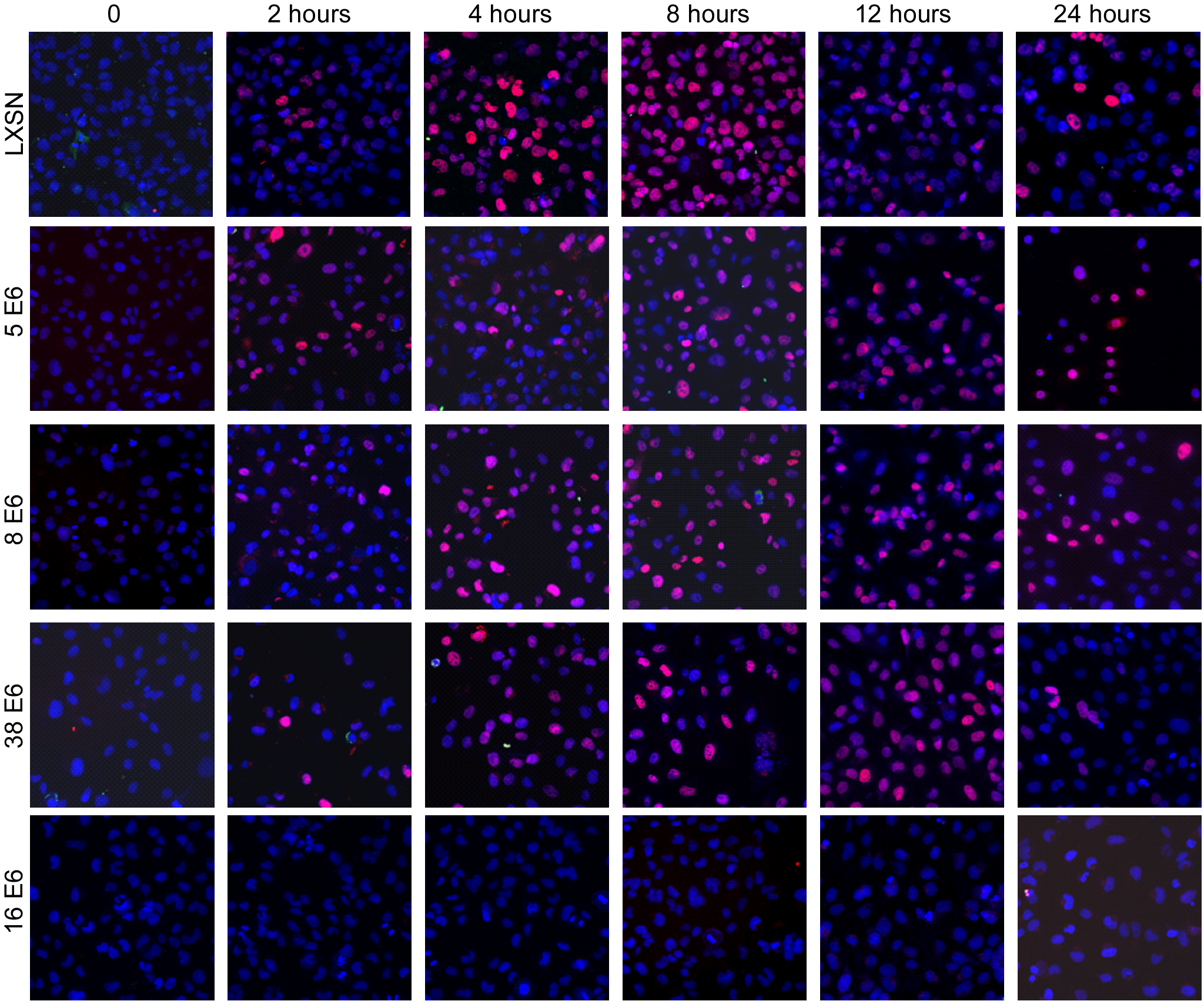

Supplement: Figure S9 — HPV β E6 expression attenuates p53 accumulation following UVB exposure. Representative fields from HT1080 cells exposed to 10 mJ/cm2 UVB with immunofluorescent detection of p53 (pink) in DAPI (blue) stained nuclei. (TIF) [file ppat.1002807.s009.tif]

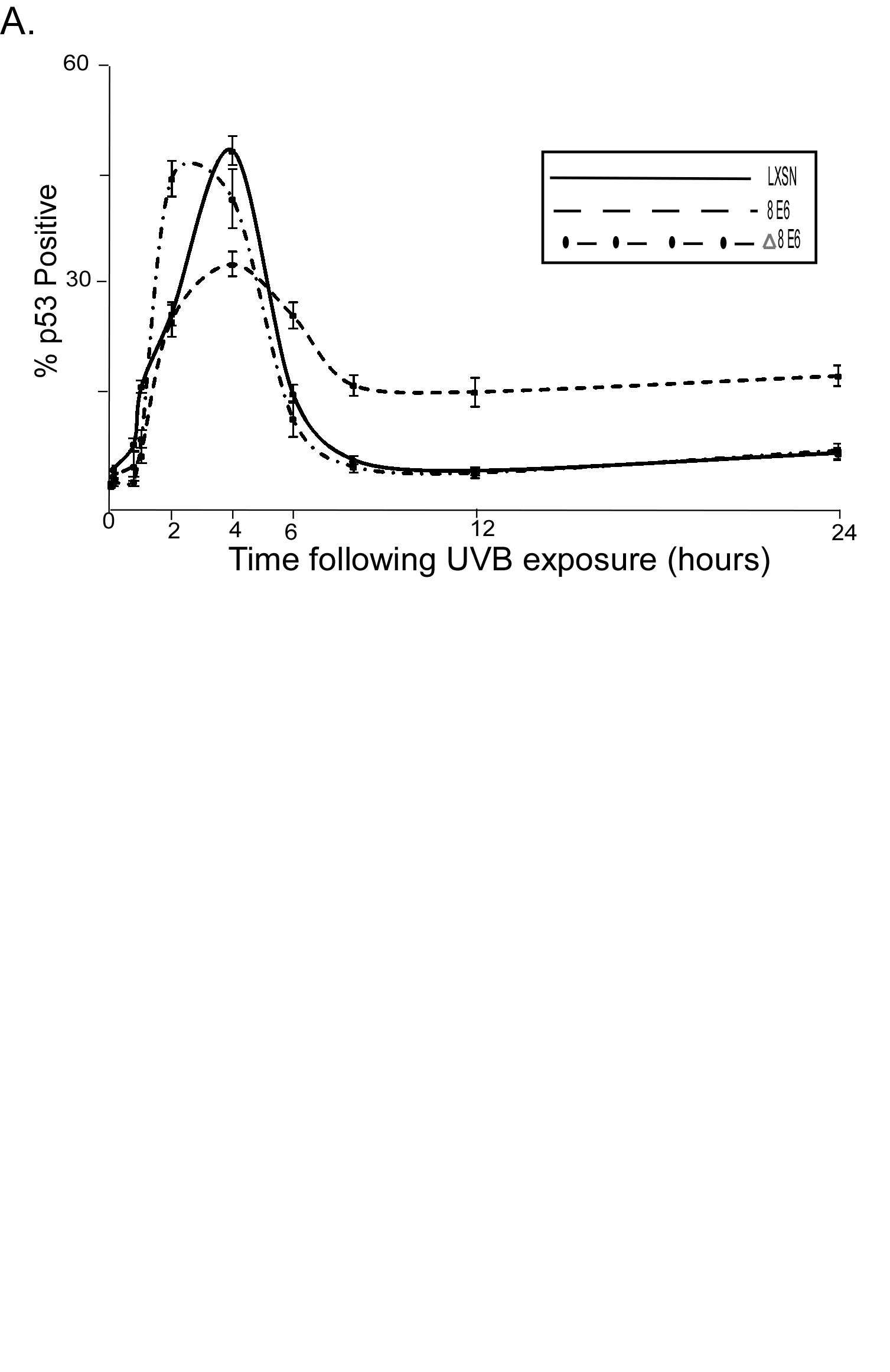

Supplement: Figure S10 — Attenuated p53 accumulation in HPV 8 E6 expressing HFK cells is dependent on p300 degradation. HFK cells were exposed to 10 mJ/cm2 UVB and immunofluorescence was used to measure the percentage of cells positive for p53 above background. (TIF) [file ppat.1002807.s010.tif]

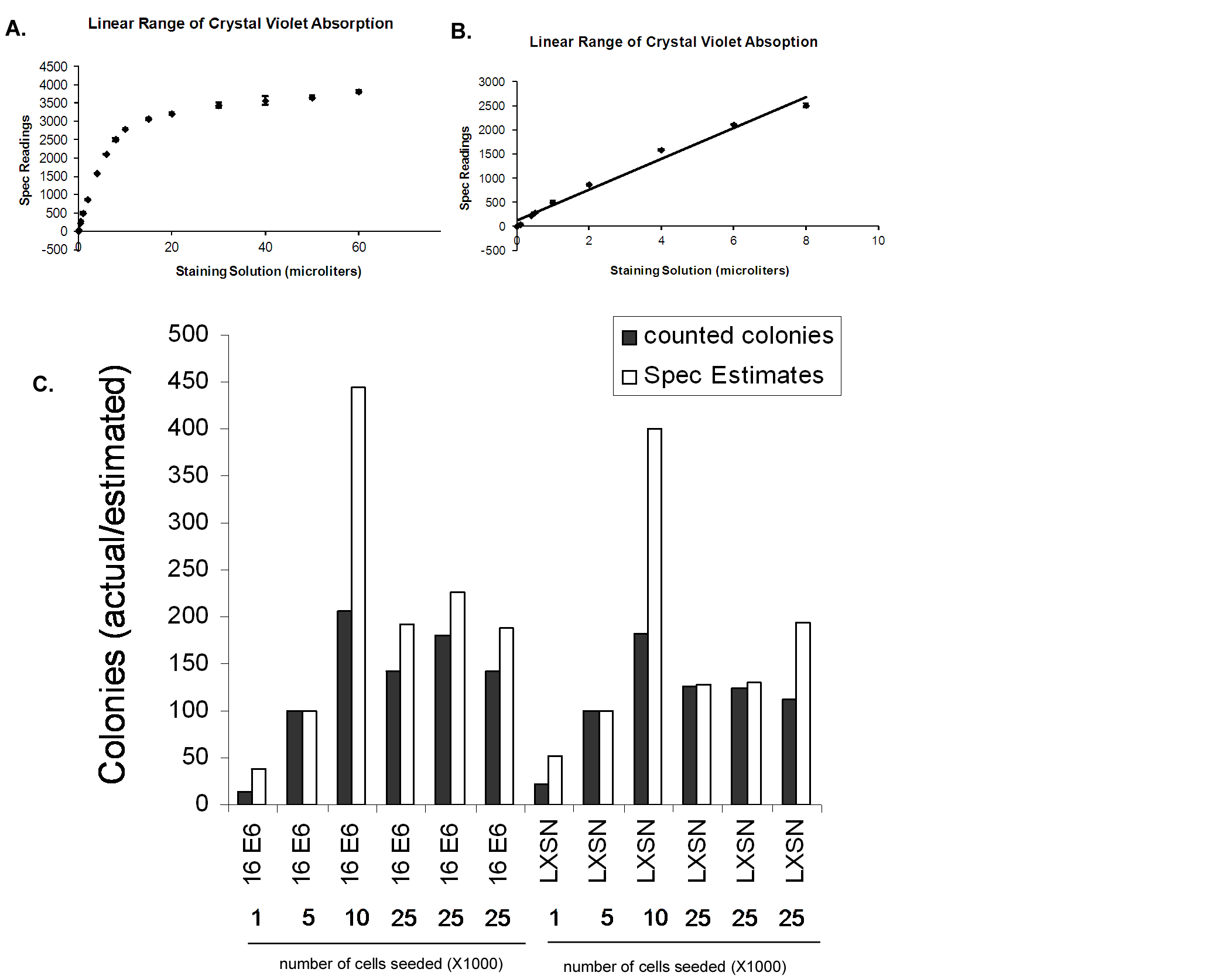

Supplement: Figure S11 — Validation of crystal violet staining accuracy. A. and B. Crystal violet absorption at 590 nm as a function of crystal violet staining solution shows a limited linear range. C. Crystal violet staining within this linear range accurately reflects colony counts for cells seeded in range from 1,000–25,000/well of a 24 well plate and grown for three days. (TIF) [file ppat.1002807.s011.tif]
